# Economical-environmental-technical optimal power flow solutions using a novel self-adaptive wild geese algorithm with stochastic wind and solar power

**DOI:** 10.1038/s41598-024-54510-1

**Published:** 2024-02-19

**Authors:** Pavel Trojovský, Eva Trojovská, Ebrahim Akbari

**Affiliations:** Department of Mathematics, Faculty of Science, University of Hradec Králové, Rokitanského 62, 500 03 Hradec Králové, Czech Republic

**Keywords:** Economical-environmental-technical dispatch problem, Electrical networks, Renewable energy sources (RESs), Optimal power flow (OPF), Self-adaptive wild geese algorithm (SAWGA), OPF optimization functions, Energy science and technology, Mathematics and computing

## Abstract

This study introduces an enhanced self-adaptive wild goose algorithm (SAWGA) for solving economical-environmental-technical optimal power flow (OPF) problems in traditional and modern energy systems. Leveraging adaptive search strategies and robust diversity capabilities, SAWGA distinguishes itself from classical WGA by incorporating four potent optimizers. The algorithm's application to optimize an OPF model on the different IEEE 30-bus and 118-bus electrical networks, featuring conventional thermal power units alongside solar photovoltaic (PV) and wind power (WT) units, addresses the rising uncertainties in operating conditions, particularly with the integration of renewable energy sources (RESs). The inherent complexity of OPF problems in electrical networks, exacerbated by the inclusion of RESs like PV and WT units, poses significant challenges. Traditional optimization algorithms struggle due to the problem's high complexity, susceptibility to local optima, and numerous continuous and discrete decision parameters. The study's simulation results underscore the efficacy of SAWGA in achieving optimal solutions for OPF, notably reducing overall fuel consumption costs in a faster and more efficient convergence. Noteworthy attributes of SAWGA include its remarkable capabilities in optimizing various objective functions, effective management of OPF challenges, and consistent outperformance compared to traditional WGA and other modern algorithms. The method exhibits a robust ability to achieve global or nearly global optimal settings for decision parameters, emphasizing its superiority in total cost reduction and rapid convergence.

## Introduction

### Motivation and incitement

The safe, secure, and inexpensive functioning of the electrical network is referred to as the optimum power flow (OPF), and this is made possible by properly configuring the system's control variables. According to the mathematical formulation, OPF is a static, nonlinear, large-scale, restricted issue with many discrete and continuous decision parameters. OPF is a vital instrument for electrical networks' cheaper and safer functioning^1,2^. OPF is used in the power system to achieve various goals, including lowering overall generating costs of thermal units (Ths), improving voltage stability, minimizing network losses, cutting CO2 emissions, and preserving the best settings for control variables^3,4^. This method is often restricted by a number of requirements that must be met, including the different technical limitations, transmission line capacity, bus voltage, ability of the power generator, and several physical limitations^5^.

Power plants using fossil fuels are the focus of the traditional OPF issue. Renewable energy sources (RESs) have proliferated over the last 20 years due to factors like rising demand, the urgent need to decrease the emissions of greenhouse gases, favorable pricing for RESs, and triggering and deregulation of the electrical energy market^6^. The most significant alternatives to fossil fuels for power production seem to be wind and solar energy^7,8^. The use of improved wind power generating units (WTs) and solar photovoltaic power units (PVs), which lower the cost of system installations, has permitted the rapidly expanding RESs use^9^. Additionally, it might be claimed that solar photovoltaic and wind turbine generating systems are tried-and-true technology^10,11^.

Based on economic indicators like the cost of fuel used to produce electrical energy in energy production systems, quality indicators of produced electrical energy, and system and transmission network losses, the performance of the electrical network operation may be significantly impacted by the locations, quantity, with technological features of RESs^12,13^. Due to its intermittent power-generating characteristics, adding several RESs into an electrical network increases the difficulty of OPF^10,14^. Recent research has focused on finding a strong, fast, and simple optimization algorithm, considering renewable energies in the electrical networks, multi-objective OPF (MOOPF) problems, and considering various objective functions to the OPF problems^15^.

### Literature review

In recent literature, numerous methods of incorporating RESs into OPF and different solution methodologies for the OPF issue have been proposed. Most scholars explored bio-inspired optimization met heuristics for OPF settings in recent studies. To tackle OPF in the electrical networks with RESs, Ullah et al. developed a combination of gravitational search algorithm (GSA) and an improved particle swarm optimization (PSO)^16^, the coronavirus herd immunity optimizer (CHIO)^17^ for the technical-environmental-economical dispatch problem in the two standards IEEE electrical networks. The five goals of this investigation were total fuel expenses, active losses, pollution level, stability, and variation of the network’s voltage. Using the analytical hierarchy process and a weighted collect method, the multi-objective OPF was reduced to a normalized one-objective OPF. The outcomes showed that CHIO outperforms the other EETD problem-solving methodologies. Elattar concentrated on applying modified moth swarm optimization (MMSO) to mathematically model the OPF with a hybrid power and heat system through stochastic WT, and the resulting methodology was applied to the IEEE 30-bus electrical network with different test settings. In contrast to previous algorithms, RESs into OPF and the proposed technique to address it generated successful optimal solutions^18^. The OPF issue was modeled by a modified colliding bodies optimization (ICBO)^19^, and applications were made using 16 case studies on three IEEE standard electrical networks to evaluate the effectiveness and resilience of ICBO. A new, improved chaotic PSO for solving MOOPF in a test system with RESs suggested in^20^, that this PSO, compared to recent algorithms, generated superior optimal solutions^20^. In^21^, the dynamic OPF (DOPF) has been optimized by a new enhanced honey bee mating algorithm while taking into account the valve-point effects (VPEs) in the 14, 30, and 118-bus standard electrical networks. Salkuti used a new glowworm swarm algorithm (GSA) that was successfully executed to resolve an MOOPF in the IEEE systems incorporating a WT under various operating circumstances^22^. A more effective improved manta ray foraging algorithm (IMOMRFO)^23^, twenty-four benchmark tasks of varying sorts and degrees of difficulty were used to examine the effectiveness of the created approach and compare it to competing methods. The analysis's findings demonstrated that IMOMRFO produced competitive results on several MOOPFs and identified the optimum results in the recent studies for MOOPFs that arise in practice^23^. Using thermal with RESs, Kathiravan et al. studied OPF using a flower pollination algorithm (FPA). In various test situations, FPA has been examined on Indian utility 30-bus and IEEE 30-bus electrical networks^24^. The suggested solution strategy for the OPF issues has been applied to the new IEEE 30-bus electrical network using a hybrid PSO-GWO algorithm, which combines PSO with grey wolf optimization (GWO)^25^. The hybrid PSO-GWO method performed well in comparison to other algorithms, according to simulation findings, and showed that it may be a good option for solving OPF issues^25^. With adjustable WT and PV energy systems, Duman et al. solved the OPF issue using the differential evolutionary (DE) PSO (DEPSO). For testing the method under various goal functions, IEEE 30-, 57-, and 118-bus electrical networks have been studied^26^. In^27^, a new hybrid firefly-bat algorithm (HFBA-COFS) directed to handle the strictly-constrained MOOPF, which the proposed method increased the original system's capacity for population diversity and global exploration of the three different IEEE test systems to demonstrate the significant benefits of the HFBA-COFS method, the HFBA-COFS algorithm was capable of producing high-quality optimal solutions, which is crucial for realizing the secure and efficient operation of massive electrical networks. In a different research, they suggested using FACTS devices to address the OPF issue. They used a modified chaotic PSOGSA to account for the uncertainty of wind energy systems^28^. A unique adaptive Gaussian teaching–learning-based optimization (TLBO) (AGTLBO) that enhanced the performance of traditional TLBO and addressed the OPF issue was proposed in^29^. The results demonstrated that it met the heuristics described in the recent studies compared to modern optimization. The AGTLBO was more efficient and successful. TLBO augmented with Lévy mutation (LTLBO)^30^ was examined, assessed, and compared to other approaches using the IEEE 30-bus and IEEE 57-bus electrical networks with various OPF functions. A modified population external optimization method (CMOPEO) was suggested by Chen et al. as an improved optimizer to OPF with RESs, and CMOPEO was evaluated on the IEEE 30-bus electrical network for several test scenarios^31^. The goal function of the slime mold algorithm (SMA)^32^ was the system's total cost, which included a penalty cost for underestimating RESs and a reserve cost for overestimation. Algeria's DZA 114-bus and IEEE 30-bus electrical networks were used to assess SMA performance. Four optimization strategies were compared to the SMA. According to the overall simulation findings, SMA outperformed the other analyzed algorithms over a variety of function landscapes. The 5-bus and new IEEE 30-bus electrical networks with and without unified power flow controller (UPFC) were used to evaluate the performance of bat optimization algorithm (BA)-based OPF as an effective and robust solution^33^. The performance of the turbulent flow of a water-based optimizer (TFWO)^34^ to OPF was demonstrated by a comparison of the statistical indices, convergence trend, and optimal solutions to modern optimizers in recent studies^35,36^. The study's findings led the authors to conclude that TFWO was better and more successful in solving OPF optimization issues. Compared to other well-known algorithms, it had higher convergence rates and made significant technological and financial advancements. The suggested TFWO for the large-scale tested system decreases the range from 4.6% to 33.12%. The proposed solution technique resulted in a more competitive solution for the evaluated system with a notable improvement in the techno-economic aspects. Three separate target functions in OPF, including reducing overall operating costs, carbon pollution with tax, and power losses, were taken into consideration by the chaotic bonobo optimizer (CBO)^37^. To demonstrate the potency and advantage of CBO to arrive at the best result, CBO was tested on two standard IEEE electrical networks. The founded optimal solutions demonstrated the effectiveness and dependability of CBO for solving OPF using stochastic RESs, a hybrid DE, symbiotic organisms search (SOS)^38^, etc.

In the realm of optimal power flow (OPF) solutions, the landscape has witnessed a surge in innovative methodologies, each striving to address the complex challenges posed by integrating renewable energy sources (RESs) into electrical networks. The advantages and disadvantages inherent in the current state-of-the-art optimization methods employed for OPF can be summarized as follows:

#### Advantages of state-of-the-art methods in OPF

1. *Versatility in Problem Solving*:
*Advantage*: Many state-of-the-art algorithms demonstrate adaptability and effectiveness in addressing specific challenges, such as technical-environmental-economical dispatch problems, making them versatile solutions.

2. *Improved Performance Metrics*:
  *Advantage*: Several methodologies exhibit superior performance in optimizing key metrics, including total fuel expenses, active losses, pollution levels, network stability, and voltage variations, showcasing their effectiveness in enhancing system efficiency.

3. *Optimal Solutions for Specific Scenarios*:
  *Advantage*: Certain algorithms excel in solving particular OPF scenarios, such as hybrid power and heat systems with stochastic renewable sources like wind turbines (WTs), providing tailored solutions for specific applications.

#### Disadvantages of state-of-the-art methods in OPF

1. *Limited Scope of Application*:
  *Disadvantage*: Some algorithms may have a narrow focus, limiting their applicability to specific IEEE electrical networks or certain types of OPF issues.

2. *Reduced Versatility*:
  *Disadvantage*: While effective for targeted challenges, certain methodologies might lack the versatility needed to address a broad spectrum of OPF problems, potentially hindering their widespread adoption.

3. *Limited Evaluation on Broader OPF Issues*:
  *Disadvantage*: Some algorithms may have limited evaluations beyond specific OPF scenarios, leaving uncertainties about their performance in addressing broader optimization challenges.

#### Contribution and paper organization

Building upon the current state-of-the-art, our work introduces a novel self-adaptive wild goose algorithm (SAWGA) to tackle the OPF problem in a modified IEEE 30-bus electrical network with stochastic RESs, including photovoltaic (PV) and WT units. With the suggested SAWGA method and a few additional optimization techniques, we attempted to solve OPF in a modified IEEE 30-bus and 118-bus electrical networks^9^, including stochastic RESs in this work. The suggested SAWGA algorithm aimed to enhance the power of basic WGA's exploitation and exploration to resolve various operational test cases of OPF, including stochastic RESs.

We have accomplished this by carefully choosing the control settings and algorithm coefficients. Lognormal and Weibull probability distribution functions were applied to stochastic RES irradiation conditions of the systems, respectively. The obtained optimal solutions by SAWGA's five evaluation items, which had been used for various OPF functions, were compared to those from the original WGA, thermal exchange optimization algorithm (TEO)^39^, grasshopper optimization (GOA)^40^, Harris hawks optimization (HHO)^41^, and honey badger algorithm (HBA)^42^, which have been recently reported.

The key contributions of our study include:

1. We present SAWGA, an enhanced version of the wild goose algorithm (WGA), demonstrating superior performance in real-world optimization problems, particularly in OPF scenarios with high difficulty levels and multiple locally optimal solutions.
2. Our proposed SAWGA method is tailored to address various OPF challenges in a modified IEEE 30-bus electrical network, accounting for the complexities introduced by stochastic RESs.
3. We conduct a thorough evaluation of SAWGA against benchmark algorithms, including thermal exchange optimization algorithm (TEO), grasshopper optimization (GOA), Harris hawks optimization (HHO), and honey badger algorithm (HBA), in addition to the original WGA. The evaluation encompasses multiple OPF optimization functions, considering diverse power generation operating situations and scenarios, such as voltage deviation, emission, and network losses.

Through these contributions, our study advances the field of bio-inspired optimization for OPF, offering a novel and efficient solution for practical applications in electrical networks with renewable energy integration.

The remains of this research have been structured as follows. OPF, with its optimization functions in the electrical networks with stochastic RESs, is presented in "Formulation of OPF in the electrical network with stochastic RESs" section. Models for RES generation power are shown in "Power and uncertainty models in the PVs and WTs" section. Two sub-sections of "The proposed algorithm" section each introduce the WGA algorithm and the suggested SAWGA approach. The experimental research conditions and the criteria considered are presented in "SAWGA for solving the different OPF problems with stochastic wind and solar power" section of the article. Finally, "Multi-objective SAWGA (MOSAWGA) for solving the different classical OPF problems" section provides the study's findings and recommendations for further research.

## Formulation of OPF in the electrical network with stochastic RESs

Traditional OPF issue was identified as an essential and vital challenge to analyze, design, and manage the power grids and energy production and transmission networks that strive to achieve the affordable and lowest cost of energy production and transmission With the condition of complying with the various stipulations and different demands, and with inequality and equality limitations of the electrical energy production and transmission system and network^9,43^. Solving this problem with various optimization functions and considering stochastic RESs such as WTs and PVs through a new modified algorithm called self-adaptive wild geese algorithm (SAWGA) has been the main goal of this research. The problem's suggested solution and its broad organization are represented below. The definition of the standard OPF issue is as follows^10^.

Minimalize:

1 $${f}_{obj}\left(x,u\right)$$

Subject to:

2 $$\begin{array}{c}a\left(x,u\right)=0,\\ b\left(x,u\right)\le 0,\end{array}$$

when ${f}_{obj}\left(x,u\right)$ is the OPFs’ optimization function, $u$ and $x$ show the independent and dependent decision parameters and the equality and inequality constraints have been shown through $a(x, u)$ and $b(x, u)$, respectively.

### Dependent parameters

The dependent parameters in OPF have been shown in Eq. (3)^10^

3 $$x=\left[{P}_{T{h}_{1}},{V}_{L},{Q}_{Th},{Q}_{WS},{Q}_{PV}\right],$$

where ${P}_{T{h}_{1}}$ shows the slack generator's active power, ${V}_{L}=\left[{V}_{{L}_{1}},\ldots,{V}_{{L}_{NPQ}}\right]$ shows the PQ buses' voltage values, ${Q}_{Th}=\left[{Q}_{T{h}_{1}},\ldots,{Q}_{T{h}_{NTHG}}\right]$, ${Q}_{WS}=\left[{Q}_{W{S}_{1}},\ldots,{Q}_{W{S}_{NW}}\right]$, and ${Q}_{PV}=\left[{Q}_{P{V}_{1}},\ldots,{Q}_{P{V}_{NPV}}\right]$ are the reactive power of Ths, WTs, and PVs, and ${S}_{L}=\left[{S}_{{L}_{1}},\ldots,{S}_{{L}_{NTL}}\right]$ shows the network lines’ transition power; *NPQ*, *NTHG*, *NW*, *NPV*, and *NTL* show the numbers of the test power system's PQ buss, Ths, WTs , PVs, and network lines.

### Independent decision parameters

These parameters of OPF have been shown follows^10^

4 $$u=\left[{P}_{Th},{P}_{WS},{P}_{PVS},{V}_{G}\right].$$

If ${V}_{G}=\left[{V}_{{G}_{1}},\ldots,{V}_{{G}_{NG}}\right]$ denotes the values of all generator buses’ voltage including PVs, Ths, WTs, and also, ${P}_{Th}=\left[{P}_{T{h}_{2}},\ldots,{P}_{T{h}_{NTHG}}\right]$, ${P}_{PVS}=\left[{P}_{PV{S}_{1}},\ldots,{P}_{PV{S}_{NPV}}\right]$, and ${P}_{WS}=\left[{P}_{W{S}_{1}},\ldots,{P}_{W{S}_{NW}}\right]$ denote the active power of Th, PV, WT units excluding the slack generator, respectively. The number of Th, PV, WT units is known as $NG, NPV,$ and $NW,$ respectively.

### Modeling of the test electrical network

This part of the study models how Th, PV, and WT power generation units may be integrated into contemporary power systems. Table 1 lists the specifications of the studied IEEE 30-bus electrical network^9^.

**Table 1:** The specifications of the test electrical network.

Characteristics	Size	Information
Branches	41	^44^
Buses	30	^44^
Slack generators	1	Bus: 1
Ths	3	Buses: 1, 2 and 8
PVs	1	Bus: 13
WTs	2	Buses: 5 and 11
Decision parameters	11	Output real power of the Ths, WTs and PVs (5 numbers); ${V}_{G}$ (6 numbers)
VAR (volt amperes reactive) sources	2	Buses: 10 and 24
Tap-changers	4	Branches: 11, 12, 15 and 36
${V}_{L}$	24	[0.95–1.05] p.u
Sum reactive and real power demands	–	126.2 MVAr, 283.4 MW

#### OPF model of Ths

According to the produced output active power, Eq. (5) describes the traditional OPF function in Ths as a quadratic objective function. Also, Eq. (6) contains the OPF model for Ths that includes VPEs, where ${r}_{i}$ and ${p}_{i}$ stand for the VPEs specifications while ${o}_{i}$, ${n}_{i}$, and ${m}_{i}$ are the OPF function specifications for the *i*th Th generator^10,45^.

5 $$CF\left({P}_{Th}\right)=\sum_{i=1}^{NTHG}\left({m}_{i}+{n}_{i}{\cdot {P}_{Th}}_{i}+{o}_{i}{\cdot P}_{T{h}_{i}}^{2}\right),$$

6 $${CF}_{1}\left({P}_{Th}\right)=\sum_{i=1}^{NTHG}\left({m}_{i}+{n}_{i}{\cdot {P}_{Th}}_{i}+{o}_{i}{\cdot P}_{T{h}_{i}}^{2}+\left|{p}_{i}\cdot {{\sin}}\left({r}_{i}\cdot \left({P}_{TH{h}_{i}}^{min}-{{P}_{Th}}_{i}\right)\right)\right|\right).$$

#### Pollution level model with carbon tax ${{\varvec{C}}}_{{\varvec{t}}{\varvec{a}}{\varvec{x}}}$

Mathematically, Eq. (7)^9,10^ describes how to calculate the overall emission value from heat-producing units utilizing fossil fuel. Additionally, a carbon tax model was taken into account owing to the increasing threat of global warming, and the emission cost value is determined through the addition ${C}_{tax}$ to the overall emission value that has been given in Eq. (8). In this case, CE and FE stand in for the emission costs, and sum emissions, respectively^9,10^.

7 $${F}_{E}=\sum_{i=1}^{NTHG}\left(0.01\cdot \left({\sigma }_{i}+{\beta }_{i}\cdot {{P}_{Th}}_{i}+{\tau }_{i}{\cdot P}_{T{h}_{i}}^{2}\right)+{\omega }_{i}\cdot {e}^{{\mu }_{i}\cdot {P}_{T{h}_{i}}}\right),$$

8 $${C}_{E}={F}_{E}{\cdot C}_{tax},$$

where ${\sigma }_{i}$, ${\tau }_{i},$ ${\omega }_{i}$, ${\beta }_{i}$, and ${\mu }_{i}$ are the emission characteristics of the *i*th Th generator.

#### Prohibited operating zones (POZs)

**Figure 1:**
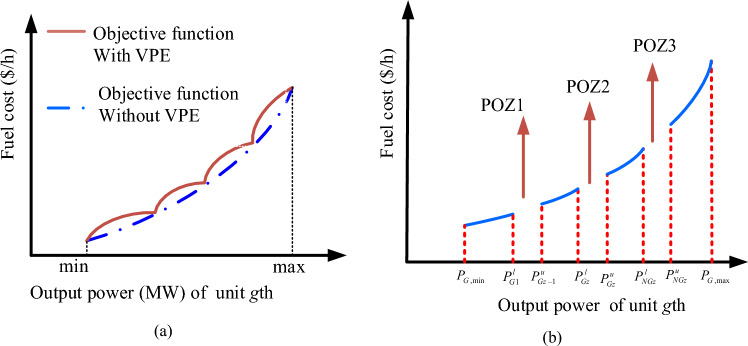
OPF objective function curves: (**a**) without and with VPEs, (**b**) with POZs.

The following characteristics of a Th generator that uses fossil fuel and has several POZs^38^:

9 $$\begin{array}{c}{P}_{T{h}_{i}}^{min}\le {P}_{T{h}_{i}}\le {P}_{T{h}_{i},1}^{L},\\ {P}_{T{h}_{i},Z-1}^{U}\le {P}_{T{h}_{i}}\le {P}_{T{h}_{i},Z}^{L}\\ {P}_{T{h}_{i},{z}_{i}}^{U}\le {P}_{T{h}_{i}}\le {P}_{T{h}_{i}}^{max},\end{array}, Z={2,3},\dots ,{z}_{i},$$

where $Z$ shows the number of POZs, ${z}_{i}$ shows the sum of POZs, ${P}_{T{h}_{i},Z}^{L},$ and ${P}_{T{h}_{i},Z-1}^{U}$ indicate the lower and upper bounds of the $(Z - 1)$ th POZ of the *i*th unit. Figure 1 shows the specification curves of the OPF objective function without and with VPEs (a), and with the POZs (b) of the Th generators.

#### Direct objective function models of RESs include PVs and WTs

A linear function of planned power may be used to represent a wind energy source's direct cost model^9,10^. Where $D{C}_{{WP}_{i}}$, ${wsh}_{i}$, and ${P}_{{WS}_{i}}$ stand for the *i*th wind power system's planned power, the direct objective function of WT, and the direct objective function specification.

10 $$D{C}_{{WP}_{i}}={P}_{{WS}_{i}}\cdot C{F}_{{WP}_{i}}={P}_{{WS}_{i}}\cdot {wsh}_{i}.$$

Equation (11) makes a model for the direct objective function of PV. Where $D{C}_{{PV}_{i}}$, ${pvsh}_{i}$, and ${{P}_{PVS}}_{i}$ have been recognized as the direct objective function of PV, the direct objective function specification, and the planned output power of ${PV}_{i}$ ^9,10^.

11 $$D{C}_{{PV}_{i}}={{P}_{PVS}}_{i}\cdot C{F}_{{PV}_{i}}={{P}_{PVS}}_{i}\cdot {pvsh}_{i}.$$

#### Uncertainty objective function models of PVs and WTs

The uncertain objective function models of PVs and WTs are characterized as the little-known and underestimation scenarios of these studied energy sources. The uncertainty objective function models of WTs are described in Eqs. (12) and (13)^9,10^.

12 $$O{C}_{WP,i}={C}_{Ow,i}\cdot \left({P}_{{WS}_{i}}-{P}_{wr,i}\right)={C}_{Ow,i}{\int }_{0}^{{P}_{WS,i}}\left({P}_{{WS}_{i}}-{p}_{w,i}\right){f}_{w}\left({p}_{w,i}\right)d{p}_{w,i},$$

13 $$U{C}_{WP,i}={C}_{Uw,i}\cdot \left({P}_{wr,i}-{P}_{{WS}_{i}}\right)={C}_{Uw,i}{\int }_{{P}_{WS,i}}^{{P}_{wr,i}}\left({p}_{w,i}-{P}_{{WS}_{i}}\right){f}_{w}\left({p}_{w,i}\right)d{p}_{w,i},$$

where $U{C}_{WP,i}$ and $O{C}_{WP,i}$ are the underestimation and overestimation objective function values, ${C}_{Ow,i}$ and ${C}_{Uw,i}$ have been defined as the uncertainty objective function specification. Also, ${P}_{{WS}_{i}}$ and ${P}_{wr,i}$ are the available and rated power of ${WT}_{i}$. A proper technique in Refs.^9,10^ had been applied to achieve the comprehensive formulation of the uncertainty objective functions of a PV, with the over- and under-estimation specifications ${C}_{WP,i}$ and $U{C}_{WP,i}$, have been given in Eq. (3):

14 $$\begin{aligned}O{C}_{PV,i}&={C}_{Opv,i}\cdot \left({P}_{PVS,i}-{P}_{PVav,i}\right)={C}_{Opv,i}\cdot {f}_{PV}\left({P}_{PVav,i}<{P}_{PVS,i}\right)\\ &\cdot \left[{{P}_{PVS}}_{i}-E\left({P}_{PVav,i}<{P}_{PVS,i}\right)\right].\end{aligned}$$

15 $$\begin{aligned}U{C}_{PV,i}&={C}_{Upv,i}\cdot \left({P}_{PVav,i}-{{P}_{PVS}}_{i}\right)={C}_{Upv,i}\cdot {f}_{PV}\left({P}_{PVav,i}>{P}_{PVS,i}\right)\\ &\cdot \left[E\left({P}_{PVav,i}>{P}_{PVS,i}\right)-{P}_{PVS,i}\right],\end{aligned}$$

where ${C}_{Opv,i}$ and ${C}_{Upv,i}$ represent the uncertainty objective function specification, and ${P}_{PVav,i}$ is the available or output power in the $i$ th PV (i.e., $PVi$).

### OPF optimization functions

#### Fuel cost optimization function considering VPEs

Equation (16) models the OPF optimization function that includes the sum of the fuel costs for Ths considering VPEs and the cost of operation and transmission of the power of the WT and PV units^9,10^.

16 $${F}_{obj1}=C{F}_{1}\left({P}_{Th}\right)+\sum_{i=1}^{NW}\left(D{C}_{WP,i}+O{C}_{WP,i}+U{C}_{WP,i}\right)+\sum_{i=1}^{NPV}\left(D{C}_{PV,i}+O{C}_{PV,i}+U{C}_{PV,i}\right).$$

#### Fuel cost optimization function considering pollution level with ${{\varvec{C}}}_{{\varvec{t}}{\varvec{a}}{\varvec{x}}}$

This OPF problem has been modeled in Eq. (17).

17 $${F}_{obj2}={F}_{obj1}+{C}_{E}.$$

#### Fuel cost optimization function considering POZs

Equation (18), which is the main goal of the mathematical model of this OPF, has been suggested to be the conventional OPF objective function in Th generators considering POZs^9,10^.

18 $${F}_{obj3}=CF\left({P}_{Th}\right)+\sum_{i=1}^{NW}\left(D{C}_{WP,i}+O{C}_{WP,i}+U{C}_{WP,i}\right)+\sum_{i=1}^{NPV}\left(D{C}_{PV,i}+O{C}_{PV,i}+U{C}_{PV,i}\right).$$

#### Network losses

The objective function presented in Eq. (19) may be used to explain the minimization of the network power losses^9,10^.

19 $${F}_{obj4}={P}_{loss}=\sum_{n=1}^{NTL}{G}_{s(i,j)}(n)\cdot \left({V}_{i}^{2}+{V}_{j}^{2}-2{V}_{i}\cdot {V}_{j}{{\cos}}{\delta }_{ij}\right),$$

where ${\delta }_{ij}$ shows the difference between voltage angles in buses *i* and *j*, and ${G}_{s(i,j)}$ shows conductance in the *s*th network line between buses $i$ and $j$. Also, the voltage value at the *i*th network bus is considered by the letter ${V}_{i}$.

#### Voltage deviation (V.D.)

In the considered OPF objective function, the *V.D.* value for the developed energy networks is determined as indicated in Eq. (20)^9,10^.

20 $${F}_{obj5}=VD=\sum_{i=1}^{NPQ}\left|{V}_{{L}_{i}}-1\right|.$$

### Considered OPF's constraints

#### Equality OPF's constraints

The suggested OPF problem's equality restrictions may be formulated as given in Eq. (21) and Eq. (22)^9,10^:

21 $${P}_{Gi}-{P}_{Di}-{V}_{i}\sum_{j=1}^{{N}_{bus}}{V}_{j}\cdot \left({B}_{ij}{{\sin}}\left({\delta }_{i}-{\delta }_{j}\right)+{G}_{ij}{{\cos}}\left({\delta }_{i}-{\delta }_{j}\right)\right)=0,$$

22 $${Q}_{Gi}+{Q}_{SHi}-{Q}_{Di}-{V}_{i}\sum_{j=1}^{{N}_{bus}}{V}_{j}\cdot \left({G}_{ij}{{\sin}}\left({\delta }_{i}-{\delta }_{j}\right)-{B}_{ij}{{\cos}}\left({\delta }_{i}-{\delta }_{j}\right)\right)=0,$$

where ${P}_{Gi}$ shows the output power of the *i*th unit, ${P}_{Di}$ shows the demanded power of the *i*th load bus, the ${Q}_{Gi}$ shows the output reactive power of the *i*th generator, and ${Q}_{SHi}$ shows the output reactive power of the *i*th parallel reactive power compensator. ${Q}_{Di}$ is the demanded reactive power of the *i*th load bus and ${N}_{bus}$ indicates the number of network buses. The transmission line's conductance and susceptance values are denoted by the letters ${G}_{ij}$ and ${B}_{ij}$.

#### Inequality OPF's constraints

- Generator limits

As indicated in Eq. (23), there are down and up restrictions on the output power levels as well as the voltage magnitudes of the Ths, WTs, and PVs^9,10^.

23 $$\begin{array}{cc}{P}_{T{h}_{i},min}\le {P}_{T{h}_{i}}\le {P}_{T{h}_{i},max},& i={1,2},\dots ,NTHG,\\ {P}_{{WS}_{i},min}\le {P}_{{WS}_{i}}\le {P}_{{WS}_{i},max},& i={1,2},\dots ,NW,\\ {P}_{{PV}_{i},min}\le {P}_{{PV}_{i}}\le {P}_{{PV}_{i},max},& i={1,2},\dots ,NPV,\\ {Q}_{T{h}_{i},min}\le {Q}_{T{h}_{i}}\le {Q}_{T{h}_{i},max},& i={1,2},\dots ,NTHG,\\ {Q}_{{WS}_{i},min}\le {Q}_{{WS}_{i}}\le {Q}_{{WS}_{i},max},& i={1,2},\dots ,NW,\\ {Q}_{{PV}_{i},min}\le {Q}_{{PV}_{i}}\le {Q}_{{PV}_{i},max},& i={1,2},\dots ,NPV,\\ {V}_{{G}_{i},min}\le {V}_{{G}_{i}}\le {V}_{{G}_{i},max},& i={1,2},\dots ,NG .\end{array}$$

- Security constraints

Each PQ bus' voltage magnitude value must fall within certain bounds, and each transmission line's apparent power value may be constrained by its maximum capacity. Where ${S}_{{L}_{i},max}$ and ${S}_{{L}_{i}}$ indicate the maximum and apparent power levels in the $i$ th line; ${V}_{{L}_{i},min}$ and ${V}_{{L}_{i},max}$ represent the minimum and maximum voltage levels of the *i*th PQ bus^9,10^.

24 $$\begin{aligned} &{S}_{{L}_{i}}\le {S}_{{L}_{i},max} {\text{for}} i={1,2},\dots ,NTL,\\ &\quad {V}_{{L}_{i},min}\le {V}_{{L}_{i}}\le {V}_{{L}_{i},max}\quad{\mathrm{for}}\; i={1,2},\dots ,NPQ.\end{aligned}$$

The SCOPFs’ objective function in the studied electrical network, including Ths, PVs, and WTs, is in Eq. (25)^46–48^.

25 $$J={f}_{obj}\left(G,H\right)+{\lambda }_{VPQ}\sum_{i=1}^{NPQ}{\left({V}_{{L}_{i}}-{V}_{{L}_{i}}^{lim}\right)}^{2}+{\lambda }_{Pslack}\cdot {\left({P}_{T{h}_{1}}-{P}_{{Th}_{1}}^{lim}\right)}^{2}+{\lambda }_{QTH}\sum_{i=1}^{NTHG}{\left({Q}_{T{h}_{i}}-{Q}_{{Th}_{i}}^{lim}\right)}^{2}+{\lambda }_{QWS}\sum_{i=1}^{NW}{\left({Q}_{{WS}_{i}}-{Q}_{{WS}_{i}}^{lim}\right)}^{2}+{\lambda }_{QPV}\sum_{i=1}^{NPV}{\left({Q}_{{PV}_{i}}-{Q}_{{PV}_{i}}^{lim}\right)}^{2}+{\lambda }_{SL}\sum_{i=1}^{NTL}{\left({S}_{{L}_{i}}-{S}_{{L}_{i}}^{lim}\right)}^{2},$$

where ${\lambda }_{VPQ}$, ${\lambda }_{Pslack}$, ${\lambda }_{QTH}$, ${\lambda }_{QWS}$, ${\lambda }_{QPV}$, and ${\lambda }_{SL}$ represent the penalty factors^9,10^ and “limit values’’ ${V}_{{L}_{i}}^{lim}, {P}_{{Th}_{1}, }^{lim}{Q}_{{Th}_{i}}^{lim},$ ${Q}_{{WS}_{i}}^{lim},$ ${Q}_{{PV}_{i}}^{lim},$ and ${S}_{{L}_{i}}^{lim}$ are defined in the same way as described in the following identity

$${x}^{lim}=\left\{\begin{array}{cc}x,& {x}^{min}\le x\le {x}^{max};\\ {x}^{max},& x>{x}^{max};\\ {x}^{min},& x<{x}^{min}.\end{array}\right.$$

## Power and uncertainty models in the PVs and WTs

Weibull probability density function (PDF), as stated in Eq. (26), identifies the wind speed distribution, where $\psi ,$, $\xi ,$ and ${v}_{w}$ indicate the scale factor, and the shape factor, the wind speed, respectively^9,10^.

26 $${f}_{v}\left({v}_{w}\right)=\frac{\xi }{\psi } {\left(\frac{{v}_{w}}{\psi }\right)}^{\xi -1}{e}^{-{\left(\frac{{v}_{w}}{\psi }\right)}^{\xi }}.$$

**Figure 2:**
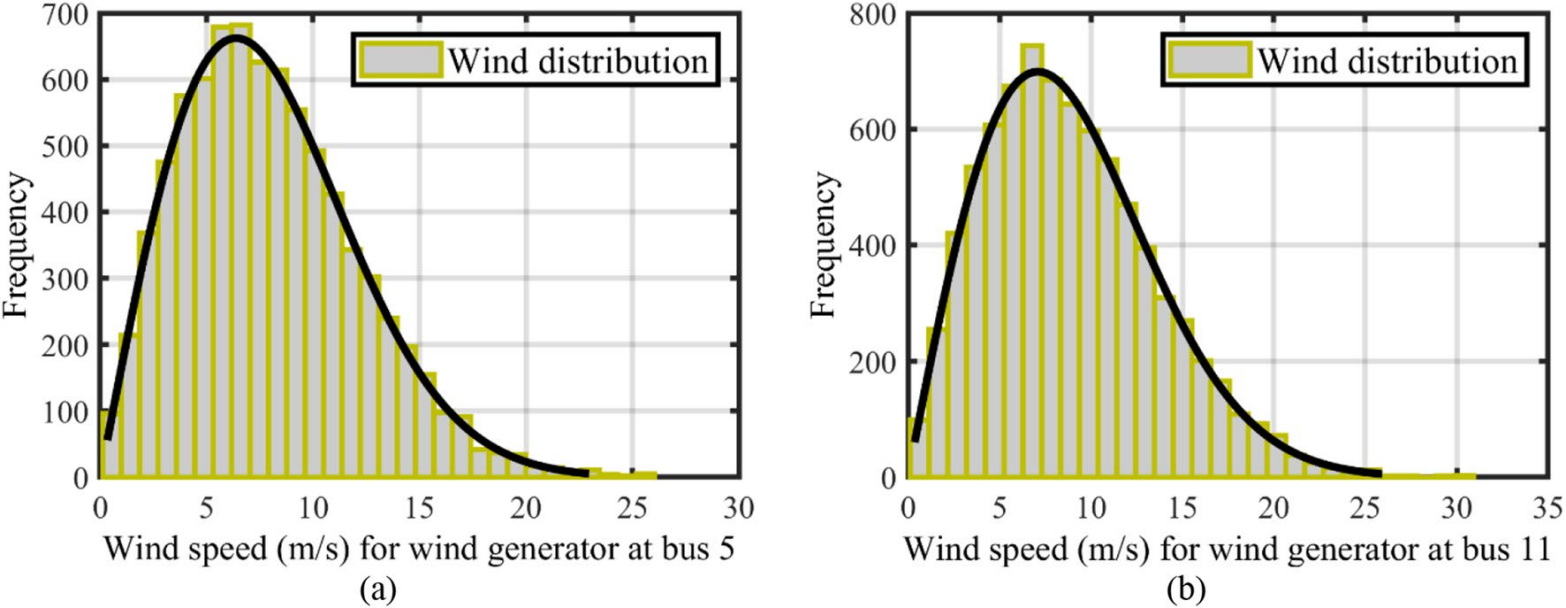
Wind frequency distributions for wind speed: (**a**) WT_1_ at bus 5, and (**b**) WT_2_ at bus 11^9^.

Weibull fittings’ outcomes in wind frequency distributions are shown in Fig. 2^9^. They were derived using a Monte Carlo simulation with 8000 iterations^9,10^. WTs’ output power has been modeled as in Eq. (27):

27 $${p}_{W}\left({v}_{w}\right)=\left\{\begin{array}{cc}0,& {{v}_{w,out}<v}_{w }{<v}_{w,in};\\ \frac{{v}_{w}-{v}_{w,in}}{{v}_{w,r}-{v}_{w,in}}\cdot {p}_{wr},& {{v}_{w,in}\le v}_{w}\le {v}_{w,r};\\ {p}_{wr},& {{v}_{w,r}\le v}_{w}\le {v}_{w,out},\end{array}\right.$$

where the rated power, cut-in, cut-out, and rated wind speeds are denoted by ${p}_{wr}$, ${v}_{w,in}$, ${v}_{w,out}$, and ${v}_{w,r}$, respectively, according to wind speeds, a wind farm's electricity is divided into distinct portions, as shown in Eq. (27). The probability values are shown between Eqs. (28) to (30) in these sections^9,10^.

28 $${f}_{w}\left({p}_{w}\right)\left\{{p}_{w}=0\right\}=1-{\text{exp}}\left(-{\left(\frac{{v}_{w,in}}{\psi }\right)}^{\xi }\right)+{\text{exp}}\left(-{\left(\frac{{v}_{w,out}}{\psi }\right)}^{\xi }\right) ,$$

29 $${f}_{w}\left({p}_{w}\right)\left\{{p}_{w}={p}_{wr}\right\}={\text{exp}}\left(-{\left(\frac{{v}_{w,r}}{\psi }\right)}^{\xi }\right)-{\text{exp}}\left(-{\left(\frac{{v}_{w,out}}{\psi }\right)}^{\xi }\right),$$

30 $${f}_{w}\left({p}_{w}\right)=\frac{\xi \cdot \left({v}_{w,r}-{v}_{w,in}\right)}{{\psi }^{\xi }{p}_{wr}}\cdot {\left({v}_{w,in}+\frac{{p}_{w}}{{p}_{wr}}\left({v}_{w,r}-{v}_{w,in}\right)\right)}^{\xi -1}\cdot {\text{exp}}\left(-{\left(\frac{{v}_{w,in}+\frac{{p}_{w}}{{p}_{wr}}\left({v}_{w,r}-{v}_{w,in}\right)}{\psi }\right)}^{\xi }\right).$$

Table 2 shows the PDF components of the PVs and WTs. Wind speeds for units had been selected as ${v}_{w,out}=25$ m/s, ${v}_{w,r}= 16$ m/s, and ${v}_{w,in}= 3$ m/s, respectively^9,10^, and with selected rated power equal to 3 MW.

**Table 2:** Specifications of the WT and PV units.

WT_1_			WT_2_			PV	
${P}_{wr}$	Turbine’s number	Weibull variables	${P}_{wr}$	Turbine’s number	Weibull variables	Lognormal parameters	Rated power (${P}_{PVrate}$)
75 MW	25	$\xi = 2, \psi = 9$	60 MW	20	$\xi = 2, \psi = 10$	$\xi = 6,\Omega = 0.6$	50 MW

The Lognormal PDF was used to characterize the generation power of PV like a mathematical model of solar irradiation. According to Eq. (31) and Eq. (32) ^9,10^, it is possible to identify the generation power and likely function of PV mathematically.

31 $${f}_{{G}_{pv}}\left({G}_{pv}\right)=\frac{1}{{G}_{pv} \cdot\Omega \sqrt{2\pi }} {\text{exp}}\left(\frac{-{\left({\text{ln}}{G}_{pv}-\xi \right)}^{2}}{2{\Omega }^{2}}\right)\quad{\mathrm{for}}\; {G}_{pv}>0,$$

where $\Omega$ and $\xi$ show the standard deviation and mean of the Lognormal PDF^9^, as shown in Table 2.

**Figure 3:**
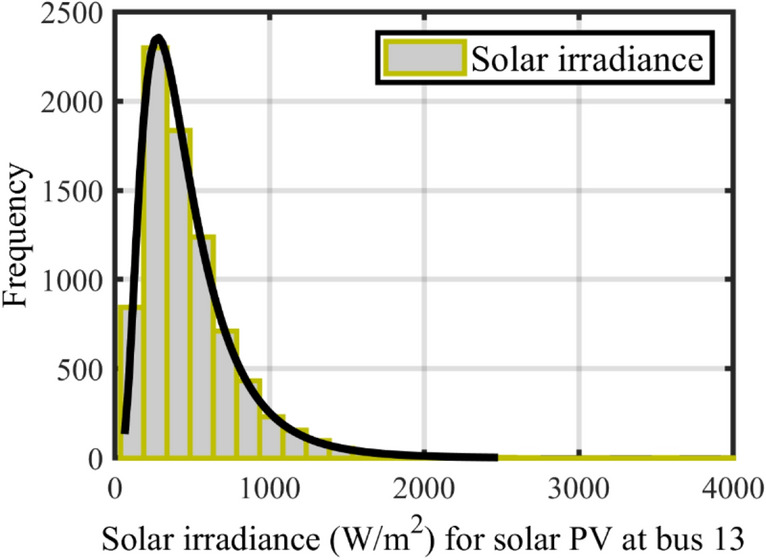
The Lognormal PDF solar irradiance distribution of PV^9^.

**Figure 4:**
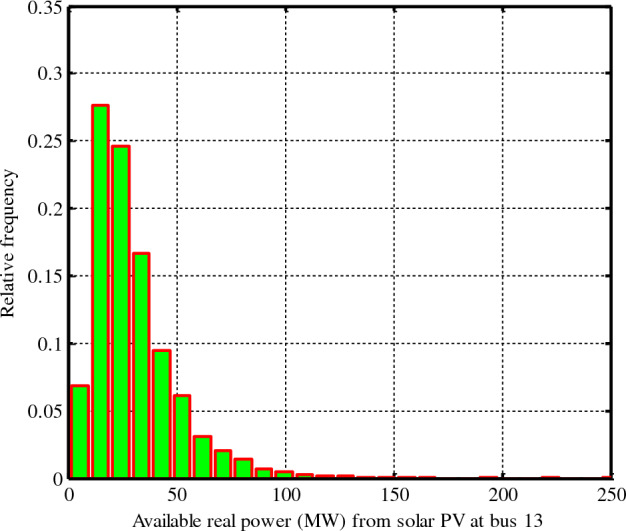
Output power (MW) distribution of PV^9^.

By an 8000-generation Monte Carlo simulation, Fig. 3 gives the frequency distribution and lognormal probability for solar irradiation^9^.

32 $${P}_{P{V}_{0}}=\left\{\begin{array}{cc}{P}_{PVrate}\cdot \frac{{G}_{pv}}{{G}_{pv}\cdot {R}_{C}} ,& 0<{G}_{pv}<{R}_{C};\\ {P}_{PVrate}\cdot \frac{{G}_{pv}}{{G}_{pvstd}},& {G}_{pv}\ge {R}_{C} ,\end{array}\right.$$

where ${G}_{pvstd}$, ${G}_{pv}$ and ${P}_{PVrate}$ are the standard solar irradiance level, the solar irradiance’s probability level, and the rated power of PV, respectively, that have been chosen equal to 800 W/m^2^ and 50 MW at bus 13. ${R}_{C}$ has been set equal to 120 W/m^2^. The generation power of PV has been shown via the histogram in Fig. 4^9^. The line shows the anticipated amount of electricity power that this PV will provide to the electrical network. It's important to keep in mind that the value of the anticipated production of solar electricity might alter.

## The proposed algorithm

In this chapter, we will focus on the construction of a new SAWGA optimizer, which is based on the improvement of the previously proposed WGA algorithm.

### Wild Goose algorithm (WGA)

In^49^, an effective method for high-dimension complicated and difficult optimization problems was introduced. Its name is the WGA, and it is inspired by the lives of wild geese, such as their altogether regular movement, breeding, and development, as well as mortality in a group of geese. In the rest of this subsection, we recap the steps of the WGA algorithm.

#### An altogether regular migration

In PSO, each wild goose has been first identified as a population member; hence, the $i$ th wild goose is equivalent to the $i$ th member with the changing location ${X}_{i}$. The migration velocity ${X}_{i}$ and individual optimal solution ${P}_{i}$ are calculated. Then, all Wild Goose populations are ranked according to their intended function, from best to worst. The wild goose migration is a collective, organized, and controlled migration that relies on power and places the upfront members so that their locations will be regularized from the most optimal solution to the worst solution (from 1 to $Np$), as $f\left({ P}_{i+2}^{Iter}\right)\le f\left({ P}_{i+1}^{Iter}\right)\le f\left({ P}_{i}^{Iter}\right)\le f\left({ P}_{i-1}^{Iter}\right)$. Equations (33) and (34) describe velocity and displacement in accordance with the altogether regular motion of the geese. According to Eq. (33), each wild goose's velocity and position changes are dependent on the speed of their front and rear goose, or $\left({V}_{i+1}^{Iter}-{V}_{i-1}^{Iter}\right)$, as well as on their locations, which have been decided through the value of the optimization function of the ordered group members $f\left({X}_{i}^{Iter}\right)$. In other words, the forefront member is the most efficient member of the wild goose since they benchmark and navigate their group as a whole as well as coordinate with the whole group. So, the migration velocity equation of the *i*th member by^49^ has the following form:

33 $$\begin{aligned}&{v}_{i,d}^{Iter+1}={r}_{1,d}\cdot {v}_{i,d}^{Iter}+{r}_{2,d}\cdot \left({v}_{i+1,d}^{Iter}-{v}_{i-1,d}^{Iter}\right)\\ &\quad+{r}_{3,d}\cdot \left({p}_{i,d}^{Iter}-{x}_{i-1,d}^{Iter}\right)+{r}_{4,d}\cdot \left({p}_{i+1,d}^{Iter}-{x}_{i,d}^{Iter}\right)\&\quad\ +{r}_{5,d}\cdot \left({p}_{i+2,d}^{Iter}-{x}_{i+1,d}^{Iter}\right)-{r}_{6,d}\cdot \left({p}_{i-1,d}^{Iter}-{x}_{i+2,d}^{Iter}\right).\end{aligned}$$

Birds in a flock always travel in the direction of the leader. The leader is in the best position globally, thus if the leader deviates, then all other birds will deviate as well. As a result, on the one hand, the best wild goose *G* serves as the group's leader-member, directing all other wild goose to their objective, and in other words, the position changes of these wild goose rely on their velocity.

Therefore, the amounts of displacement and change ${X}_{i}$ resulting from wild goose migration, i.e., ${X}_{i}^{V}$, are calculated based on the member's best local position ${P}_{i}$ and migration velocity ${V}_{i}$, as well as the member who is in front of them ${P}_{i+1}$, the group leader $G$ which has been explained in Eq. (34). The geese constantly adjust their separation from one another to maintain a safe spacing. The product of two random numbers between 0 and 1,${r}_{7,d}\cdot {r}_{8,d}$ has been utilized since the quantity is small^49^:

34 $${x}_{i,d}^{V}={r}_{7,d}\cdot {r}_{8,d}\cdot \left({v}_{i,d}^{Iter+1}+\left({p}_{i+1,d}^{Iter}+{g}_{d}^{Iter}-2{p}_{i,d}^{Iter}\right)\right)+{p}_{i,d}^{Iter}.$$

#### Search for food

The *i*th wild goose (or any other goose) moves toward its upfront wild goose ${P}_{i+1}^{Iter}$, and the upfront goose's walking and seeking for food has been modelled as its scheme. The $i$ th member imitates the $(i+1)$ th member and attempts to achieve that ${P}_{i+1}^{Iter}-{X}_{i}^{Iter}$. The wild geese's ${X}_{i}^{W}$ equation for foraging while on the move is as follows^49^

35 $${x}_{i,d}^{W}={p}_{i,d}^{Iter}+{r}_{9,d}\cdot {r}_{10,d}\cdot \left({p}_{i+1,d}^{Iter}-{p}_{i,d}^{Iter}\right).$$

#### Reproduction and evolution of wild gooses

One part of wild gooses’ living similar to other alive extant has been based on the reproduction and evolution. Its modeling has been applied similarly to that of DE algorithm (Eq. (36)), where a combination (crossover operation) between migration equation ${X}_{i}^{V}$, search for food ${X}_{i}^{W}$ has been applied. In^49^, $Cr$ size in the WGA algorithm has been considered equal to 0.5.

36 $${x}_{i,d}^{Iter+1}=\left\{\begin{array}{cc}{x}_{i,d}^{V},& {r}_{11,d}\le Cr;\\ {x}_{i,d}^{W},& \text{otherwise.}\end{array}\right.$$

#### Regular evolution, migration and death

The algorithm starts out in this phase with the maximum population ${Np}^{initial}$, and as it iterates, the weaker individuals in terms of Eq. (37) will be eliminated from the total members (death of the weaker individuals) and the act of optimization will continue until the final members in the final generation achieves its final size ${Np}^{final}$. Algorithm 1 illustrates the code for this simple and effective approach^49^.

37 $$p={\text{round}}\left(N{p}^{initial}-(N{p}^{initial}-N{p}^{final})\frac{FEs}{FE{s}_{max}}\right),$$

where ${FEs}_{max}$ shows the maximum fitness evaluations and $FEs$ shows the recent fitness evaluations.

**Algorithm 1:**
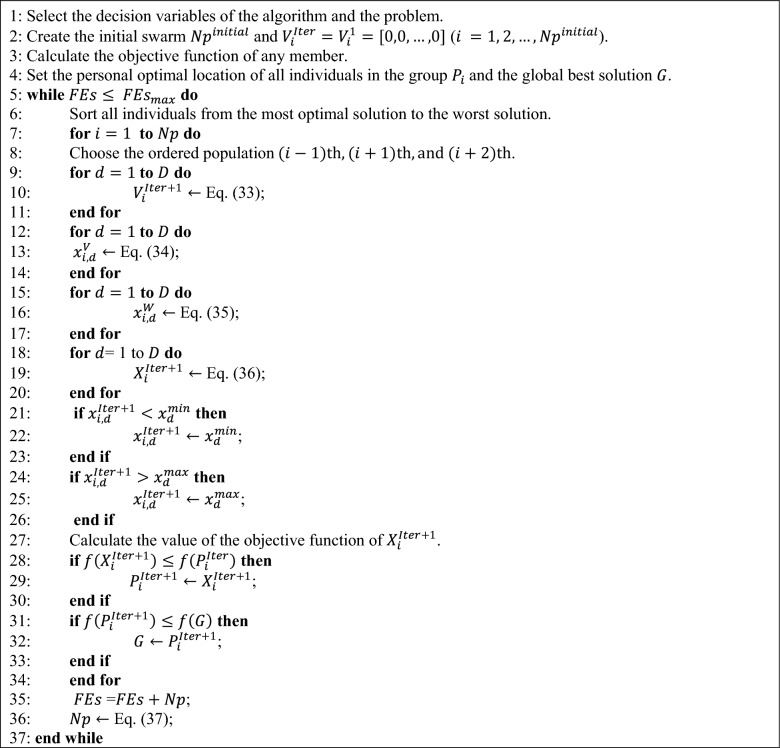
.

**Figure 5:**
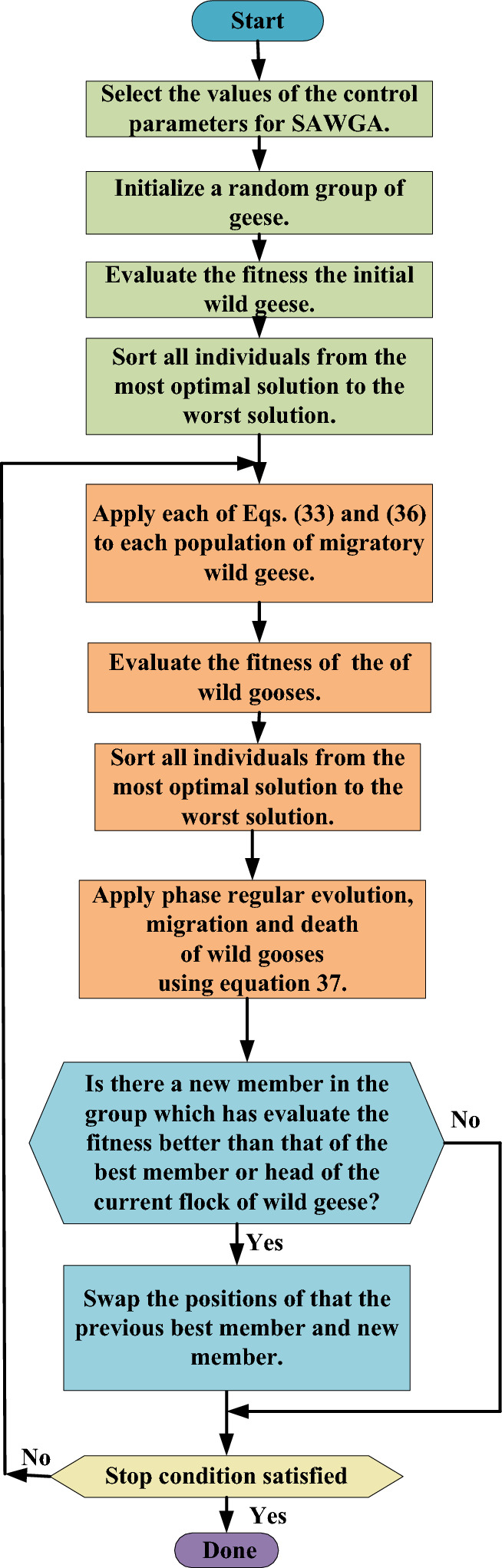
Optimization process of the proposed modified algorithm.

In addition, the optimization process of the proposed modified algorithm has been shown in Fig. 5.

### Self-adaptive decision parameters in WGA: A Self-adaptive wild geese algorithm (SAWGA)

Choosing appropriate control parameter values is typically a challenge that depends on the type of issue. Multiple optimization runs are needed since the control settings are tuned by trial and error^50^. We provide a self-adaptive technique for control settings in this section. Values for the parameter are extended to each member of the population. For Eqs. (34) to (36), we chose control-based random coefficients and modeled them for this investigation. We have recreated the equations once again and identified an appropriate connection for them, as indicated in Eqs. (38) to (40).

${\varvec{C}}{\varvec{r}}$ and ${\varvec{F}}$ are the control variables that will be modified through evolution. Both are used on an individual basis. Superior individuals result from these (encoded) control parameter values because they are more likely to live, procreate, and spread these superior parameter values to new individuals.

38 $${x}_{i,d}^{Iter+1}=\left\{\begin{array}{cc}{F}_{i}^{Iter}\cdot \left({v}_{i,d}^{Iter+1}+\left({p}_{i+1,d}^{Iter}+{g}_{d}^{Iter}-2{p}_{i,d}^{Iter}\right)\right)+{p}_{i,d}^{Iter},& C{r}_{i}^{Iter}\ge rand;\\ {p}_{i,d}^{Iter}+{F}_{i}^{Iter}\cdot \left({p}_{i+1,d}^{Iter}-{p}_{i,d}^{Iter}\right),& \begin{array}{c}\\ {\text{o}}{\text{t}}{\text{h}}{\text{e}}{\text{r}}{\text{w}}{\text{i}}{\text{s}}{\text{e}}\text{.}\end{array}\end{array}\right.$$

39 $${F}_{i}^{Iter+1}=\left\{\begin{array}{cc}0.1+ran{d}^{2}\cdot 0.9,& rand<0.1;\\ {F}_{i}^{Iter},& \begin{array}{c}\\ {\text{o}}{\text{t}}{\text{h}}{\text{e}}{\text{r}}{\text{w}}{\text{i}}{\text{s}}{\text{e}}\text{.}\end{array}\end{array}\right.$$

40 $${Cr}_{i}^{Iter+1}=\left\{\begin{array}{cc}0.4+rand\cdot 0.2,& rand<0.1;\\ {Cr}_{i}^{Iter},& {\text{otherwise}}.\end{array}\right.$$

where *rand* is a uniform random number from the interval [0, 1].

## SAWGA for solving the different OPF problems with stochastic wind and solar power

**Figure 6:**
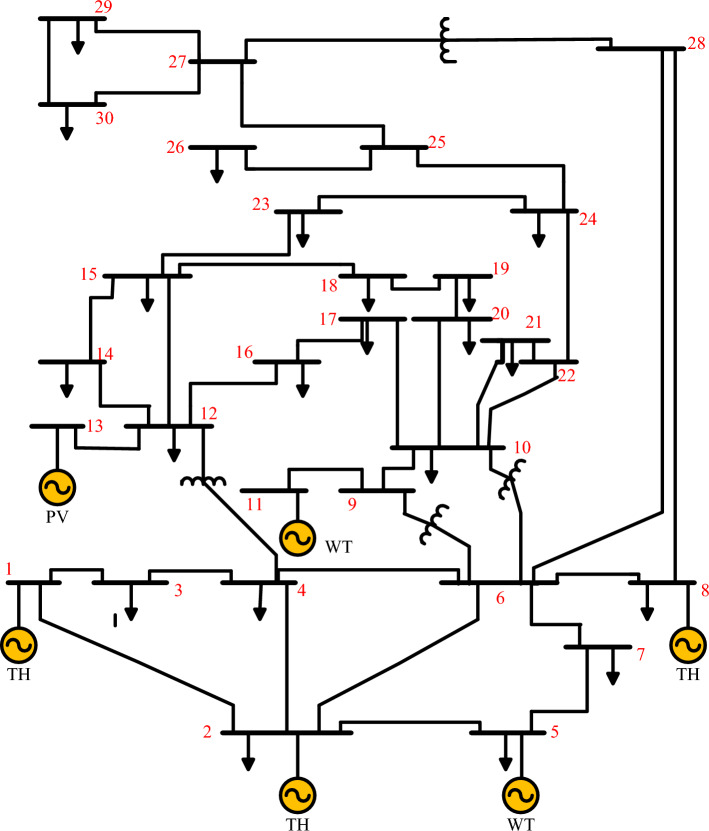
The IEEE 30-bus electrical network.

On the IEEE 30-bus electrical network, as given in Fig. 6, the WGA, GOA, TEO, HHO, HBA, and SAWGA approaches were investigated to resolve an OPF issue, including PV and VT units. Additionally, Table 3 provides the control settings for the optimization techniques that were collected from references. The system parameters for the IEEE 30-bus electrical network have been shown in Table 4 and were derived from references^3,9,10,51^.

**Table 3:** The control variables of the optimization techniques.

Technique	Control variables
HHO	${E}_{0} \in [-1, 1]$
TEO	$Np= 60$
	$$TMs = 3$$
	$${c}_{1}={c}_{2}=1$$
GOA	$Np= 60$
	Attractive length size $=$ 1.5
	Value of attraction $= 0.5$
	$${C}_{max} = 1$$
	$${C}_{min} = 0.00004$$
HBA	$Np= 60$
	$$\beta = 6$$
	$$C = 2$$
WGA	${NP}_{min}=30, {NP}_{max}=90$
SAWGA	${NP}_{min}=30, {NP}_{max}=90$
	Initial values ($Iter=1$): ${F}_{i}^{1}={(rand)}^{2},$ ${Cr}_{i}^{1}=0.5$ for $i={1,2},\dots ,{NP}_{max}.$

**Table 4:** The pollution level and cost factors of Th units in the IEEE 30-bus electrical network.

Thermal generator	Bus no.	*n*	*m*	*R*	*p*	*o*	*σ*	*μ*	*ω*	*τ*	*B*	POZs
Th_1_	1	2	0	0.037	18	0.00375	4.091	6.667	0.0002	6.49	− 5.554	[55 66] [80 120]
Th_2_	2	1.75	0	0.038	16	0.0175	2.543	3.333	0.0005	5.638	− 6.047	[21 24] [45 55]
Th_3_	8	3.25	0	0.045	12	0.00834	5.326	2	0.002	3.38	− 3.55	[25 30]

Table 5 displays the underestimation, overestimation, and direct cost factors for PVs and WTs. The load flow framework shown in MATPOWER^51,52^ has been applied to this OPF, including Ths, PVs, and WTs. For the optimization functions of the suggested OPF, all optimization methods were performed 30 times to statistically analyze the obtained optimal solutions. The optimization functions of the suggested OPF have been optimized in accordance with the different scenarios that follow.

**Table 5:** The cost factors of the PVs and WTs of the electrical network.

WT_1_				WT_2_				PV			
Bus no	${C}_{Uw,1}$	${C}_{Ow,1}$	$wsh,1$	Bus no	${C}_{Uw,2}$	${C}_{Ow,2}$	$wsh,2$	Bus no	${C}_{Upv,1}$	${C}_{Opv,1}$	$pvsh,1$
5	1.5	3	1.60	11	1.5	3	1.75	13	1.5	3	1.60

- Case 1: OPF considering total cost in Th units considering VPEs, PVs, and WTs.
- Case 2: OPF considering total cost in Th units considering emission and carbon tax, and PVs and WTs.
- Case 3: OPF considering total cost in Th units considering POZs, PVs, and WTs.
- Case 4: OPF considering power losses of the electrical network.
- Case 5: OPF considering the voltage deviation (*V.D.*) of the electrical network.

### Case 1: Total cost considering VPEs of Th units

**Figure 7:**
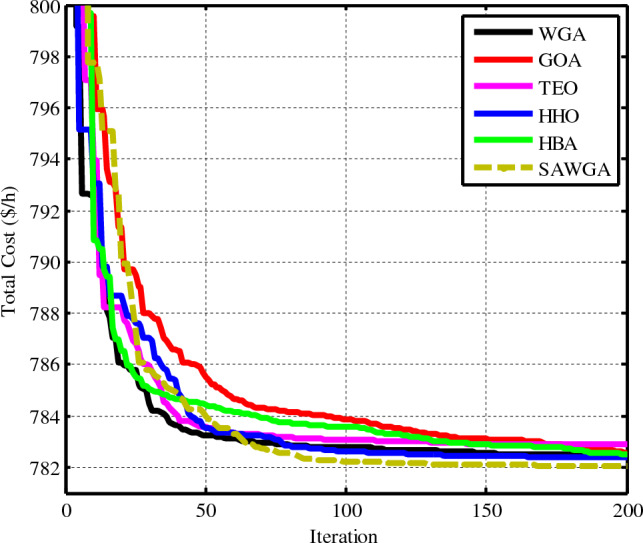
Convergences of the optimization techniques for Case 1.

OPF’s objective function, considering VPEs of Th units, as well as the cost function of the PVs and WTs, are used in Case 1 to optimize for lowering the overall cost. In this instance, produced electric power from all various kinds of generation units employed in the redesigned electrical network are optimized to reduce the basic power cost to its absolute lowest. Table 6 lists the outcomes of all factors that were ideally determined, including reactive powers, total cost, decision parameters, and other variables—values the best, worst, average, and Std. for the algorithms and the suggested SAWGA approach after 30 separate runs are shown in Table 6. The simulation findings of this case demonstrate the efficacy of SAWGA. SAWGA offers a quick convergence trend and improved results quality compared to the traditional WGA, GOA, TEO, HHO, and HBA optimization approaches for OPF. SAWGA obtained the lowest overall power cost of 782.0238 $/h among all used algorithms. The convergences of used OPF optimization approaches have been given in Fig. 7.

**Table 6:** The obtained optimal decision parameters for Case 1.

Variables	WGA	GOA	TEO	HHO	HBA	SAWGA
*P*_*Th1*_ (MW)	134.90791	134.90791	134.90791	134.90791	134.90791	134.90791
*P*_*Th2*_ (MW)	27.5394	29.1664	29.1839	29.0028	26.8694	27.7761
*P*_*WS1*_ (MW)	43.2401	44.1165	44.1506	44.0164	42.823	43.3368
*P*_*Th3*_ (MW)	10	10	10	10	10	10
*P*_*WS2*_ (MW)	36.4904	37.2214	37.2336	37.1532	36.1747	36.5999
*P*_*PV*_ (MW)	37.1045	33.755	33.7839	34.089	38.422	36.566
*V*_*1*_ (p.u.)	1.0734	1.0718	1.0738	1.0718	1.072	1.0715
*V*_*2*_ (p.u.)	0.95	1.0569	0.9675	1.0568	1.0569	1.0565
*V*_*5*_ (p.u.)	1.0376	1.035	1.0382	1.0349	1.0348	1.0345
*V*_*8*_ (p.u.)	1.1	1.066	1.043	1.0428	1.0396	1.0464
*V*_*11*_ (p.u.)	1.0998	1.0992	1.1	1.0999	1.0999	1.0989
*V*_*13*_ (p.u.)	1.0639	1.0486	1.0631	1.0488	1.0557	1.0495
*Q*_*Th1*_ (MVAR)	16.5841	−2.22675	16.7964	−2.31606	−1.96676	−2.40918
*Q*_*Th2*_ (MVAR)	−20	11.7839	−20	11.8166	13.1232	11.7019
*Q*_*WS1*_ (MVAR)	30.1073	22.4293	30.1335	22.4292	23.2508	22.4298
*Q*_*Th3*_ (MVAR)	40	40	40	40	34.8876	40
*Q*_*WS2*_ (MVAR)	30	30	30	30	30	30
*Q*_*PV*_ (MVAR)	20.7435	15.0147	20.4621	15.0742	17.7668	15.3163
Fuelvlvcost ($/h)	437.3882	442.7895	442.8482	442.2433	435.1854	438.1692
Wind gen cost ($/h)	242.9330	248.4681	248.6292	247.8849	240.4334	243.6381
Solar gen cost ($/h)	102.1602	91.2926	91.4006	92.0410	106.9016	100.2165
Total Cost ($/h)	782.4813	782.5502	782.8780	782.3692	782.5204	**782.0238**
Emission (t/h)	1.76232	1.76193	1.76192	1.76196	1.76249	1.76226
Power losses (MW)	5.8823	5.7672	5.8599	5.7694	5.7969	5.7866
*V.D.* (p.u.)	0.49643	0.45370	0.49452	0.45400	0.46508	0.45505
Mean	782.9547	783.8471	784.2549	783.4879	783.6828	**782.1907**
Max	783.2486	785.3005	785.8745	784.6940	785.0236	**782.3373**
Std	1.04	3.62	2.95	1.76	2.45	**0.49**
Time (s)	20	25	21	15	23	18

Significant values are in bold.

### Case 2: Total cost considering emission and carbon tax in Th units

**Figure 8:**
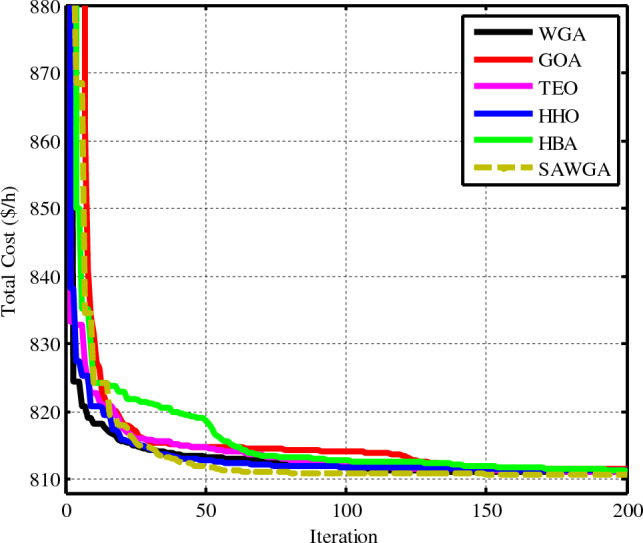
Convergences of the optimization techniques for Case 2.

By imposing a set $${C}_{tax}$$ on Th units due to their CO2 emissions, the case's goal is to reduce the electricity’s total cost^9^. The mandated $${C}_{tax}$$ is set at $20 per ton^9^. The simulation findings clearly support the idea that imposing the carbon fee would increase the amount of wind and solar energy that is incorporated into the electricity system. Similar to scenario 1, Fig. 8 compares the convergence of the SAWGA, WGA, GOA, TEO, HHO, and HBA algorithms, and Table 7 lists the best results for OPF as Table 6. SAWGA outperforms WGA and all other used approaches based on overall cost reduction and the convergence of the beast result since it achieves a minimum value of 810.7448 $/h.

**Table 7:** The obtained optimal decision parameters for Case 2.

Variables	WGA	GOA	TEO	HHO	HBA	SAWGA
*P*_*Th1*_ (MW)	123.90712	123.68049	122.98256	123.15774	123.73478	123.22507
*P*_*Th2*_ (MW)	34.1193	33.4781	31.6383	31.7003	33.6271	32.2519
*P*_*WS1*_ (MW)	46.5768	46.2435	45.2817	45.4877	46.321	45.6065
*P*_*Th3*_ (MW)	10	10	10	10.0061	10	10
*P*_*WS2*_ (MW)	39.197	38.938	38.1574	38.0722	38.9908	38.4111
*P*_*PV*_ (MW)	34.8778	36.3406	40.6216	40.3586	36.0031	39.1813
*V*_*1*_ (p.u.)	1.0709	1.0701	1.0695	1.0692	1.0708	1.0704
*V*_*2*_ (p.u.)	1.0574	1.0566	1.056	0.9567	1.0573	1.0569
*V*_*5*_ (p.u.)	1.0364	1.0356	1.0349	1.0385	1.0362	1.0357
*V*_*8*_ (p.u.)	1.0405	1.1	1.0517	1.0438	1.0404	1.0403
*V*_*11*_ (p.u.)	1.0991	1.0982	1.0999	1.0985	1.0985	1.0986
*V*_*13*_ (p.u.)	1.0551	1.0503	1.0516	1.0956	1.0555	1.0566
*Q*_*Th1*_ (MVAR)	−2.60697	−3.06275	−3.25719	11.6126	−2.62087	−2.74585
*Q*_*Th2*_ (MVAR)	12.4289	10.9399	10.6912	−20	12.3734	12.2339
*Q*_*WS1*_ (MVAR)	22.9514	22.2315	22.2344	30.5365	22.9545	22.9783
*Q*_*Th3*_ (MVAR)	35.4048	40	40	40	35.3368	35.1783
*Q*_*WS2*_ (MVAR)	30	30	30	28.9583	30	30
*Q*_*PV*_ (MVAR)	17.4826	15.5674	16.0543	25	17.6094	18.0251
Fuelvlvcost ($/h)	434.1073	431.3640	423.4359	424.0774	432.0042	426.0933
Wind gen cost ($/h)	264.1178	261.9972	255.8144	256.2487	262.4630	257.8581
Solar gen cost ($/h)	94.6553	100.2372	114.6302	113.5536	98.7041	109.2680
Emission (t/h)	0.91105	0.89932	0.86428	0.87299	0.90211	0.87627
Carbon tax ($/h)	18.221	17.9864	17.2856	17.4598	18.0422	17.5254
Total Cost ($/h)	811.1014	811.5848	811.1662	811.3395	811.2135	**810.7448**
Power losses (MW)	5.2781	5.2808	5.2817	5.3826	5.2767	5.2758
*V.D.* (p.u.)	0.46771	0.46010	0.46261	0.53792	0.46835	0.47047
Mean	811.4573	812.4095	812.3281	812.6439	812.2931	**810.8538**
Max	811.7822	813.7250	813.8394	814.0215	814.2465	**810.9647**
Std	0.66	2.06	2.61	3.82	3.23	**0.26**
Time (s)	22	30	25	23	23	20

Significant values are in bold.

### Case 3: OPF considering POZs in Th units

**Figure 9:**
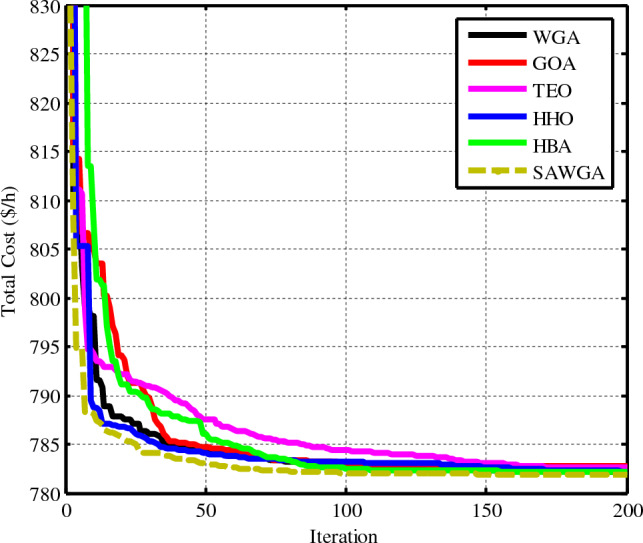
Convergences of the optimization techniques for Case 3.

According to the OPF problem stated in Eq. (9), optimization of the overall cost while taking POZs has been explored. Like instances 1 and 2, Fig. 9 compares the convergence of the SAWGA, WGA, GOA, TEO, HHO, and HBA algorithms, and Table 8 lists the optimum attained results for the reactive powers, total cost, decision parameters, and other variables. The suggested SAWGA algorithm produced a simulation result of 781.9047 $/h, which was superior to that of the WGA and other approaches.

**Table 8:** The obtained optimal decision parameters for Case 3.

Variables	WGA	GOA	TEO	HHO	HBA	SAWGA
*P*_*Th1*_ (MW)	134.90790	134.90850	134.90814	134.90809	134.90802	134.90804
*P*_*Th2*_ (MW)	28.6119	30.242	28.9972	29.0366	29.0567	28.2065
*P*_*WS1*_ (MW)	43.8509	44.8314	44.1742	43.9827	43.9828	43.6865
*P*_*Th3*_ (MW)	10	10.0193	10.0001	10.0001	10.0001	10.0003
*P*_*WS2*_ (MW)	36.9721	37.8204	37.3252	37.2498	37.1606	36.877
*P*_*PV*_ (MW)	34.9236	31.3305	33.7572	33.9922	34.063	35.4969
*V*_*1*_ (p.u.)	1.0737	1.0722	1.0719	1.0716	1.0718	1.0716
*V*_*2*_ (p.u.)	0.9548	1.0564	1.057	1.0567	1.0569	1.0567
*V*_*5*_ (p.u.)	1.0381	1.034	1.0344	1.0348	1.035	1.0349
*V*_*8*_ (p.u.)	1.0417	1.0942	1.088	1.0779	1.058	1.0879
*V*_*11*_ (p.u.)	1.1	1.0999	1.0989	1.0992	1.0983	1.098
*V*_*13*_ (p.u.)	1.064	1.0511	1.0483	1.05	1.0483	1.0488
*Q*_*Th1*_ (MVAR)	16.8049	−0.28789	−2.26905	−2.48558	−2.28611	−2.49022
*Q*_*Th2*_ (MVAR)	−20	9.71985	12.5211	11.6477	11.9056	11.8111
*Q*_*WS1*_ (MVAR)	30.2971	21.5993	21.822	22.3699	22.5011	22.634
*Q*_*Th3*_ (MVAR)	39.5107	40	40	40	40	40
*Q*_*WS2*_ (MVAR)	30	30	30	30	30	29.9958
*Q*_*PV*_ (MVAR)	20.7866	15.9995	14.9279	15.482	14.8925	15.0588
Fuelvlvcost ($/h)	440.9404	446.4787	442.2255	442.3571	442.4240	439.5956
Wind gen cost ($/h)	246.6879	253.0467	249.0266	248.0990	247.7929	245.7918
Solar gen cost ($/h)	94.5294	83.2327	91.3001	91.7759	91.9761	96.5174
Total Cost ($/h)	782.1578	782.7581	782.5522	782.2321	782.1931	**781.9047**
Emission (t/h)	1.76205	1.76174	1.76199	1.76197	1.76196	1.76217
Power losses (MW)	5.8664	5.7521	5.7619	5.7695	5.7712	5.7752
*V.D.* (p.u.)	0.49687	0.45719	0.45268	0.45669	0.45307	0.45409
Mean	782.6849	783.5542	783.6004	783.2400	783.2576	**781.9862**
Max	783.1025	784.9218	784.4879	784.6198	784.3775	**782.2004**
Std	0.95	1.76	2.07	1.65	2.38	**0.44**
Time (s)	19	22	23	17	22	20

Significant values are in bold.

### Case 4: OPF considering power losses of the electrical network

**Figure 10:**
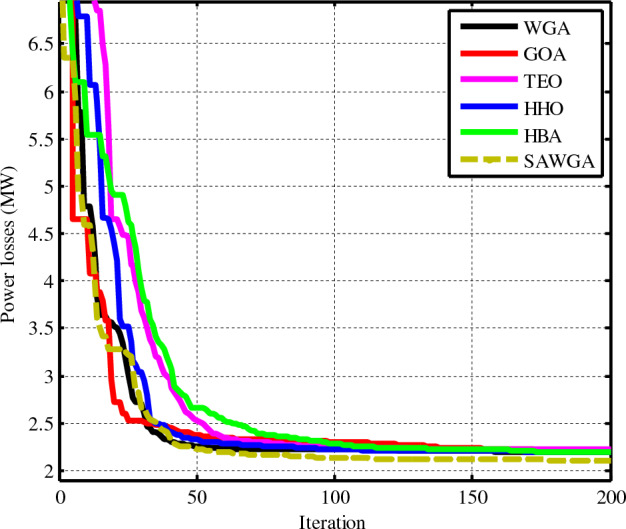
Convergences of the optimization techniques for Case 4.

In this instance, the considered algorithms suggested optimizing the power losses of the electrical network updated by utilizing PVs and WTs. The SAWGA method produced a result of 2.1037 MW for this scenario, less than the optimization results from the other methods. Also, Table 9 shows this case's findings at the simulation research's conclusion. For scenario 4, Fig. 10 compares the convergence of the SAWGA, WGA, GOA, TEO, HHO, and HBA algorithms.

**Table 9:** The obtained optimal decision parameters for Case 4.

Variables	WGA	GOA	TEO	HHO	HBA	SAWGA
*P*_*Th1*_ (MW)	50.01016	50	50	50.00520	50.01259	50.00007
*P*_*Th2*_ (MW)	30.8297	29.6365	33.5344	31.0493	31.1131	26.4672
*P*_*WS1*_ (MW)	74.9993	75	75	74.971	74.9965	74.9997
*P*_*Th3*_ (MW)	24.9873	24.995	24.995	24.979	24.9946	34.9998
*P*_*WS2*_ (MW)	59.9967	60	60	59.994	59.9868	59.9983
*P*_*PV*_ (MW)	44.7663	45.9704	45.1225	44.5924	44.4865	40.3606
*V*_*1*_ (p.u.)	1.0587	1.0558	0.9956	1.0585	1.0586	1.025
*V*_*2*_ (p.u.)	1.0535	1.0507	1.0551	1.0534	1.0534	1.0547
*V*_*5*_ (p.u.)	1.0438	1.0413	1.0455	1.0433	1.0444	1.0454
*V*_*8*_ (p.u.)	1.0481	1.0984	1.0579	1.0492	1.0486	1.0834
*V*_*11*_ (p.u.)	1.0987	1.0995	1.0983	1.0962	1.0984	1.0987
*V*_*13*_ (p.u.)	1.0608	1.0999	1.0645	1.0584	1.059	1.0628
*Q*_*Th1*_ (MVAR)	−5.14494	−7.1348	−20	−5.6223	−5.23928	−20
*Q*_*Th2*_ (MVAR)	7.05482	3.03437	19.0167	7.14925	6.3457	19.1593
*Q*_*WS1*_ (MVAR)	20.9941	19.3565	20.7818	20.2086	21.5572	20.7092
*Q*_*Th3*_ (MVAR)	37.6769	40	40	39.7903	38.5714	40
*Q*_*WS2*_ (MVAR)	30	30	30	30	30	30
*Q*_*PV*_ (MVAR)	19.483	25	20.2871	18.5296	18.8021	19.4471
Fuelvlvcost ($/h)	280.3009	276.2794	289.5516	280.9959	281.2985	306.6375
Wind gen cost ($/h)	464.6118	464.6296	464.6295	464.4749	464.5561	464.6208
Solar gen cost ($/h)	130.3026	135.3708	131.5669	129.5661	129.6609	113.4794
Total Cost ($/h)	875.2153	876.2797	885.7481	875.0370	875.5155	884.7377
Emission (t/h)	0.09832	0.09857	0.09779	0.09827	0.09826	0.09856
Power losses (MW)	2.1895	2.2019	2.2191	2.1909	2.1901	**2.1037**
*V.D.* (p.u.)	0.51452	0.58045	0.54504	0.50839	0.50964	0.54543
Mean	2.3512	3.4109	3.5379	3.8471	3.7853	**2.2447**
Max	2.5460	5.6310	6.8116	5.4972	6.0025	**2.3769**
Std	0.46	1.71	2.93	2.55	3.84	**0.09**
Time (s)	22	29	22	20	32	21

Significant values are in bold.

### Case 5: OPF considering the voltage deviation (*V.D.*)

**Figure 11:**
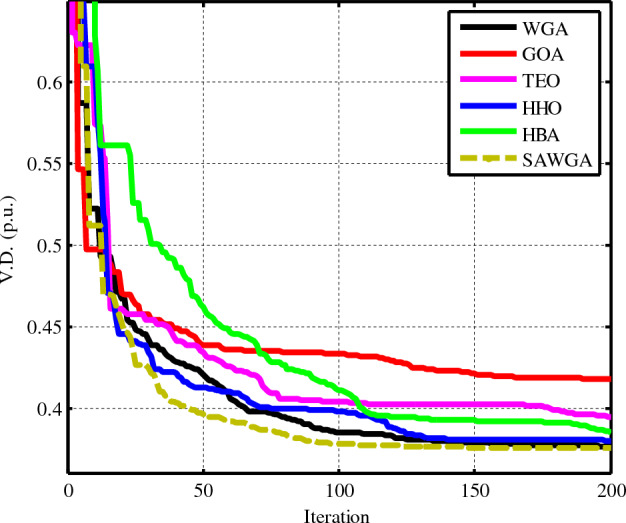
Convergences of the optimization techniques for Case 5.

In Case 5, the goal was to optimize *V.D.* of the electrical network using PVs and WTs. The outcomes of the WGA, GOA, TEO, HHO, HBA, and SAWGA algorithms were, respectively, 0.37658, 0.41849, 0.39531, 0.38066, 0.38570, and 0.37576, as shown in Table 10. Table 10 makes it quite evident that the SAWGA method produced a worse outcome than the other algorithms. Figure 11 displays the convergence patterns for all optimization techniques.

**Table 10:** The obtained optimal decision parameters for Case 5.

Variables	WGA	GOA	TEO	HHO	HBA	SAWGA
*P*_*Th1*_ (MW)	85.57691	82.95199	126.35970	115.13293	107.29577	75.29884
*P*_*Th2*_ (MW)	75.6244	57.2661	52.7137	60.8624	26.8608	80
*P*_*WS1*_ (MW)	70.0387	65.138	46.6673	65.1588	71.3622	75
*P*_*Th3*_ (MW)	33.7984	17.118	28.1129	28.0727	33.6943	35
*P*_*WS2*_ (MW)	22.6919	45.1453	24.5383	18.3631	39.0337	22.548
*P*_*PV*_ (MW)	0.5356	26.9636	12.0993	1.5673	9.8616	0
*V*_*1*_ (p.u.)	1.0584	0.9818	1.022	1.05	1.0619	1.0532
*V*_*2*_ (p.u.)	1.0931	1.0689	1.0381	1.0741	1.0959	1.0918
*V*_*5*_ (p.u.)	0.9904	0.998	1.0459	0.9965	0.9689	0.9961
*V*_*8*_ (p.u.)	1.056	1.0737	1.0595	1.0992	1.0915	1.0322
*V*_*11*_ (p.u.)	1.0961	1.0696	1.0964	1.0932	1.0992	1.1
*V*_*13*_ (p.u.)	1.0913	1.0512	1.0641	1.076	1.0583	1.0646
*Q*_*Th1*_ (MVAR)	−14.4845	−20	−20	−20	−7.86506	−20
*Q*_*Th2*_ (MVAR)	60	60	9.08326	60	60	60
*Q*_*WS1*_ (MVAR)	−23.7824	−13.0033	35	−15.6104	−30	−19.623
*Q*_*Th3*_ (MVAR)	40	40	40	40	40	40
*Q*_*WS2*_ (MVAR)	30	24.4989	30	30	30	30
*Q*_*PV*_ (MVAR)	25	21.7706	25	25	23.8183	25
Fuelvlvcost ($/h)	592.0660	443.8770	580.9108	585.8586	466.3532	585.2897
Wind gen cost ($/h)	309.4195	359.8614	219.6178	279.0695	363.9070	330.7898
Solar gen cost ($/h)	44.9266	71.9106	47.8197	45.2999	46.6644	45.1197
Total Cost ($/h)	946.4122	875.6491	848.3482	910.2280	876.9247	961.1992
Emission (t/h)	0.16822	0.15360	1.04732	0.55693	0.37526	0.13551
Power losses (MW)	4.8659	4.0261	5.9212	5.7282	4.7084	4.4532
*V.D.* (p.u.)	0.37658	0.41849	0.39531	0.38066	0.38570	**0.37576**
Mean	0.38853	0.52405	0.48743	0.45954	0.47150	**0.37925**
Max	0.39607	0.63921	0.55459	0.53006	0.51993	**0.38264**
Std	0.075	0.24	0.13	0.18	0.20	**0.015**
Time (s)	17	26	18	20	22	19

Significant values are in bold.

## Multi-objective SAWGA (MOSAWGA) for solving the different classical OPF problems

To address the multi-objective problem (MOP), we utilize the Pareto method, elaborated upon in the subsequent section:

### Pareto optimization method

In the realm of multi-objective problems (MOPs), the optimization process encompasses the simultaneous optimization of multiple independent objective functions (OFs), all while adhering to a spectrum of equality and inequality constraints. The formulation of these problems is as follows^39,40^:

41 $${{\min}}J(x,u)={\left[{J}_{1}(x,u),{J}_{2}(x,u),\ldots,{J}_{n}(x,u)\right]}^{T}.$$

The Pareto method endeavors to identify a collection of solutions that takes into account all OFs and strikes a balance among them. The ultimate outcome of this process is identifying of Pareto optimal points—solutions that are not surpassed by any other solutions. In a broader sense, solution ${X}_{1}$ is considered to dominate solution ${X}_{2}$ if the following conditions are satisfied:

42 $$\begin{aligned}\forall i\in \left\{{1,2},\ldots,n\right\},{J}_{i}({X}_{1})\le {J}_{i}({X}_{2})\\ \exists h\in \left\{{1,2},\ldots,n\right\},{J}_{h}({X}_{1})<{J}_{h}({X}_{2})\end{aligned}$$

The group of solutions not dominated by other solutions is termed “dominant solutions.” To manage the size of this set, the paper employs the fuzzy grouping method.

### Fuzzy grouping method

In certain MOPs, there is a need to standardize OFs to bring their values into a comparable range. Subsequently, the normalized values of OFs are merged. The fuzzy grouping method introduces a membership function (MF) for each OF, characterized by:

43 $${\sigma }_{{J}_{i}}(X)=\left\{\begin{array}{cc}1,& {J}_{i}\left(X\right)<{J}_{i}^{{{\min}}};\\ 0,& {J}_{i}\left(X\right)>{J}_{i}^{{{\max}}};\\ \frac{{J}_{i}^{{{\max}}}-{J}_{i}(X)}{{J}_{i}^{{{\max}}}-{J}_{i}^{{{\min}}}},& {J}_{i}^{{{\min}}}{\le J}_{i}\left(X\right)\le {J}_{i}^{{{\max}}},\end{array}\right.$$

where ${J}_{i}^{{{\min}}}$ and ${J}_{i}^{{{\max}}}$ show the minimum and maximum values of the $i$ th OF, respectively, these values are calculated by optimizing each OF as a single objective problem.

For available solutions in the stored set, the normalized value of MF is computed using (44):

44 $${N}_{\sigma }\left(i\right)=\frac{\sum_{h=1}^{n}{\phi }_{h}\cdot {\sigma }_{{J}_{h}}\left({X}_{i}\right)}{\sum_{i=1}^{q}\sum_{h=1}^{n}{\phi }_{h}\cdot {\sigma }_{{J}_{h}}\left({X}_{i}\right)},$$

where $q$ shows the number of non-dominated solutions and ${\phi }_{h}$ is the weighing factor pertaining to the $h$-th OF.

### MOSAWGA to solve multi-objective OPF problems

To solve multi-objective OPF problems using MOSAWGA, the subsequent procedure is followed:

**Table:** 

Step 1: Enter the necessary data for the algorithm and system Step 2: Embedding constraints into objective functions using the penalty functions method Step 3: Initialization of the wild goose population Step 4: Evaluate the objective function for the wild gooses Step 5: Putting non-dominated solutions in a repository Step 6: Sorting population considering the normalized values of the objective function of the previous step Step 7: Applying the altogether regular migration, search for food, and reproduction and evolution of wild gooses Step 8: Regular evolution, migration, and death Step 9: Swapping wild gooses’ positions and refreshing dominant solutions in the repository Step 10: Repeating steps 7 to 9 until finishing the total number of iterations	

### Numerical results and comparison

**Figure 12:**
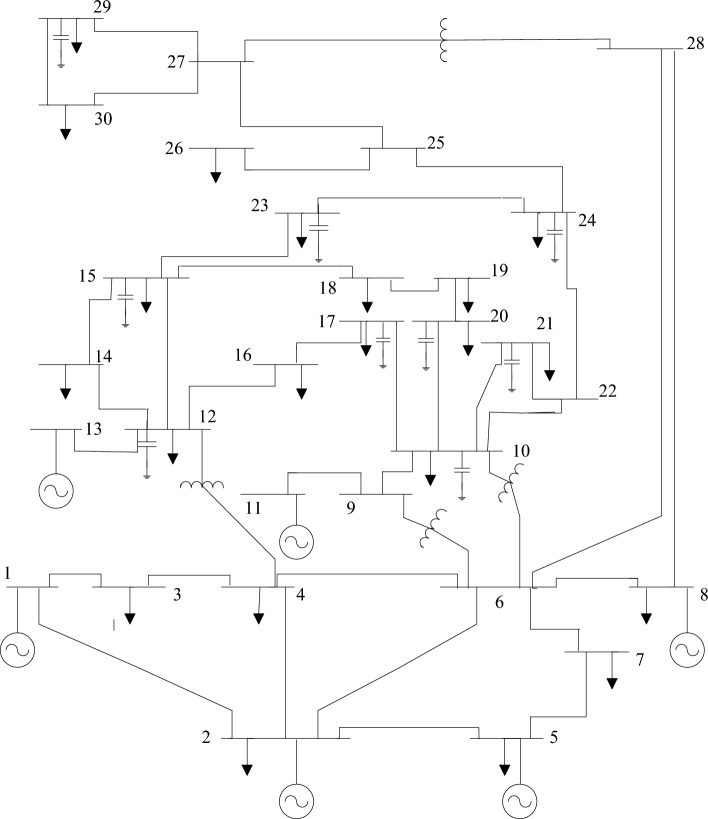
One-line representation of the IEEE 30-bus system.

To evaluate the performance and efficacy of MOSAWGA, it is examined on the IEEE 30-bus power system, demonstrated in Fig. 12^1,2,6^.

This system has six generating units located at buses 1, 2, 5, 8, 11, and 13 and four tap-adjusting transformer units at branches 6–9, 6–10, 4–12, and 28–27. The total system power demand is 2.834 p.u. with base power equal to 100 MVA base. The lower limits of voltage magnitudes of generator buses, voltage magnitudes of load buses, VAR compensation, and transformers’ tap settings are set to 0.95, 0.95, 0.0, and 0.95 p.u., respectively. Also, their higher limits are set to 1.1, 1.05, 0.3, and 1.1 p.u., respectively.

Setting the parameters of the algorithms is the same as in the fifth section. To demonstrate the efficiency of the proposed algorithm, the following three cases were taken into account:

**Case 1:** Minimization of fuel cost and real power transmission losses.

**Case 2:** Minimization of fuel cost and voltage magnitudes’ deviance (voltage profile improvement).

**Case 3:** Minimization of fuel cost and emissions.

#### Case 1: Minimization of fuel cost and real power losses

The best fuel costs and power losses of the best compromise solution (BCS) achieved by MOSAWGA and those of other algorithms are presented in Table 11. The best fuel costs and real power losses achieved by MOSAWGA are 822.0989 $/h and 5.55 MW, respectively.

**Table 11:** The best results of Case 1 by different algorithms.

Algorithm	PG1 (MW)	PG2 (MW)	PG5 (MW)	PG8 (MW)	PG11 (MW)	PG13 (MW)	Losses (MW)	Cost ($/h)
EGA–DQLF^6^	–	49.5	30.06	34.98	23.96	21.374	5.613	822.87
FPSO^17^	–	59.88	34.62	33.4	30	23.56	5.6658	847.011
NSGA-II^15^	134.5544	46.2891	32.936	30.1163	18.735	26.5392	5.7699	823.8875
MOHS^15^	118.5673	51.5253	27.855	34.9822	28.6026	27.1048	5.3143	832.6709
MOWGA	131.2	59.821	36.347	29.3022	19.2264	13.5948	6.0907	827.8943
MOSAWGA	128.23	51.6756	29.8891	35	24.4712	19.6843	5.55	822.0989

**Figure 13:**
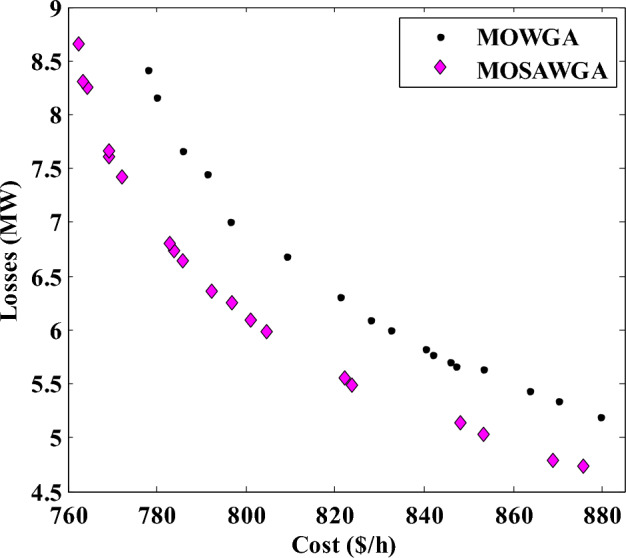
Final non-dominated solutions obtained for Case 1.

The final non-dominated solutions of different algorithms are demonstrated in Fig. 13 (only 20 points are presented).

#### Case 2: Minimization of fuel cost and voltage magnitudes deviation

Table 12 gives the summarized optimization results obtained by MOSAWGA, and Table 13 compares the best result of MOSAWGA with that of other metaheuristic methods mentioned in the introductory part of the article.

**Table 12:** Best values for decision variables and the minimum fuel costs for different cases using MOSAWGA.

Variables	Limits		Case 1	Case 2	Case 3
	Min	Max			
*P*_*G1*_ (MW)	50	200	128.23	176.22	96.43
*P*_*G2*_ (MW)	20	80	51.6756	48.9725	61.7513
*P*_*G5*_ (MW)	15	50	29.8891	21.6661	32.6168
*P*_*G8*_ (MW)	10	35	35	22.3661	34.1582
*P*_*G11*_ (MW)	10	30	24.4712	12.198	28.1513
*P*_*G13*_ (MW)	12	40	19.6843	12	35.1393
*V*_*G1*_ (p.u.)	0.95	1.1	1.1	1.0335	1.0674
*V*_*G2*_ (p.u.)	0.95	1.1	1.0879	1.0196	1.0561
*V*_*G5*_ (p.u.)	0.95	1.1	1.0624	1.0195	1.0260
*V*_*G8*_ (p.u.)	0.95	1.1	1.0713	1.0057	1.0371
*V*_*G11*_ (p.u.)	0.95	1.1	1.0881	1.0039	1.0312
*V*_*G13*_ (p.u.)	0.95	1.1	1.076	1.0107	1.0326
*T*_*6–9*_ (p.u.)	0.9	1.1	1.0729	1.0179	0.9845
*T*_*6–10*_ (p.u.)	0.9	1.1	0.9344	0.9	0.9685
*T*_*4-12*_ (p.u.)	0.9	1.1	1.0186	0.9848	0.9920
*T*_*28–27*_ (p.u.)	0.9	1.1	0.9958	0.9737	0.9616
*Qc*_*10*_ (p.u.)	0.0	0.05	0.03	0.05	0.03
*Qc*_*12*_ (p.u.)	0.0	0.05	0.03	0.03	0.04
*Qc*_*15*_ (p.u.)	0.0	0.05	0.04	0.05	0.03
*Qc*_*17*_ (p.u.)	0.0	0.05	0.05	0.0	0.03
*Qc*_*20*_ (p.u.)	0.0	0.05	0.04	0.05	0.03
*Qc*_*21*_ (p.u.)	0.0	0.05	0.05	0.05	0.03
*Qc*_*23*_ (p.u.)	0.0	0.05	0.03	0.05	0.02
*Qc*_*24*_ (p.u.)	0.0	0.05	0.05	0.05	0.03
*Qc*_*29*_ (p.u.)	0.0	0.05	0.03	0.04	0.02
Cost ($/h)			822.0989	804.35	862.923
Losses (MW)			5.55	10.0247	5.8433
*VD* (p.u.)			1.2827	0.0965	0.5027
Emission (ton/h)			0.262	0.3635	0.2244

**Table 13:** The best results of Case 2 by different algorithms.

Algorithm	*P*_*G1*_ (MW)	*P*_*G2*_ (MW)	*P*_*G5*_ (MW)	*P*_*G8*_ (MW)	*P*_*G11*_ (MW)	*P*_*G13*_ (MW)	*VD* (p.u.)	Cost ($/h)
DE^2^	183.1277	47.4435	18.7281	16.1515	11.8855	16.505	**0.1357**	**805.2619**
BBO^11^	173.4298	49.06	21.77	23.27	13.84	11.98	**0.102**	**804.9982**
MOWGA	172.4	6.3816	3.9732	3.0121	14.3573	13.0979	**0.1416**	**805.9642**
MOSAWGA	176.22	48.9725	21.6661	22.3661	12.198	12	**0.0965**	**804.35**

Significant values are in bold.

**Figure 14:**
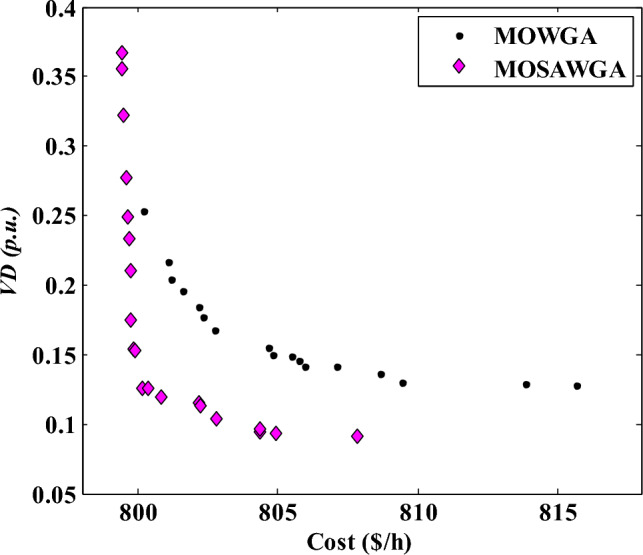
Final non-dominated solutions found for Case 2.

The Pareto optimal solutions acquired by WGA-based methods are presented in Fig. 14. It is observed in the figure that MOSAWGA gives well-distributed solutions compared with other algorithms.

#### Case 3: Minimization of fuel cost and emissions

**Figure 15:**
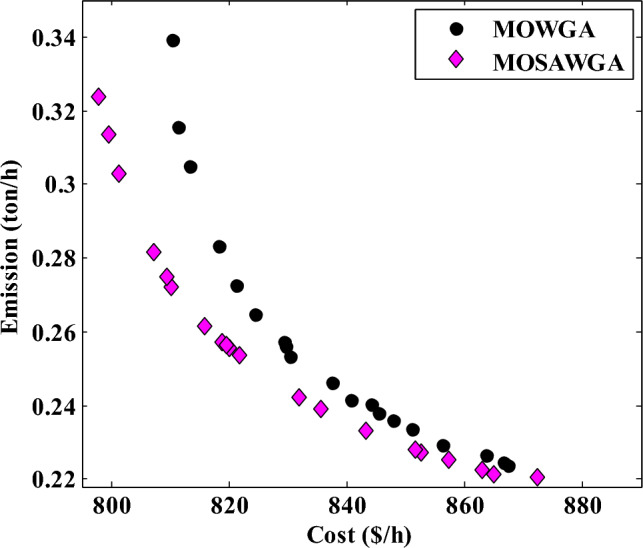
Final non-dominated solutions found for Case 3.

The best solutions found by MOSAWGA in 50 trials for Case 3 are shown in Table 12. Also, Table 14 yields a comparison of all algorithms. It is deduced by investigating these tables that MOSAWGA found a solution with fuel cost equal to 862.923 $/h and emission equal to 0.2244 ton/h, which are less than those of other algorithms. The non-dominated solutions of this case are given in Fig. 15.

**Table 14:** The best results of Case 3 by different algorithms.

Algorithm	*P*_*G1*_ (MW)	*P*_*G2*_ (MW)	*P*_*G5*_ (MW)	*P*_*G8*_ (MW)	*P*_*G11*_ (MW)	*P*_*G13*_ (MW)	Emission (ton/h)	Cost ($/h)
MSFLA^25^	97.55027	60.42367	31.6343	35	30	35.21483	**0.2247**	**867.713**
MPSO-SFLA^31^	97.11	61.19	31.47	35	30	35.11	**0.2246**	**868.372**
MOWGA	94.29	66.7893	33.8824	32.1805	27.791	33.445	**0.2245**	**866.7013**
MOSAWGA	96.43	61.7513	32.6168	34.1582	28.1513	35.1393	**0.2244**	**862.923**

Significant values are in bold.

## IEEE 118 bus test network

The effectiveness of the proposed SAWGA in addressing larger power systems is assessed using the IEEE 118-bus test system^53–64^ in the field of electrical engineering. This test network features 54 generators, 2 reactors, 12 capacitors, 9 transformers, and 186 branches. A total of 129 control variables are considered, encompassing 54 generator active powers and bus voltages, 9 transformer tap settings, and 12 shunt capacitor reactive power injections. Voltage limitations for all buses are maintained between 0.94 and 1.06 p.u. The shunt capacitors offer reactive powers from 0 to 30 MVAR, and transformer tap settings are assessed within the range of 0.90–1.10 p.u.^26^.

### Case 1: The quadratic cost function for conventional generators in OPF, excluding solar and wind energy sources, can be expressed as follows:

In Table 15, the performance of the proposed SAWGA is juxtaposed with outcomes from alternative algorithms explored in the field of electrical engineering. A comprehensive literature review also includes various techniques utilized for solving large-scale OPF problems. The comparative analysis in these tables showcases the superiority of SAWGA over other optimization methodologies in achieving optimal OPF solutions. The simulation results reveal a noteworthy reduction in cost, with SAWGA achieving a minimum cost of $129,522.5891 per hour, surpassing the outcomes produced by alternative algorithms.

**Table 15:** Optimal results for Case 1.

Method	Min	Mean	Max	Std.	Time (s)
SAWGA	**129,522.5891**	**129,528.3342**	**129,532.0716**	**5.19**	**598**
WGA	130,027.7312	130,124.9829	130,370.8430	83.85	607
FHSA^65^	132,138.3	132,138.3	132,138.3	0.0	–
ETFWO^36^	129,542.8215	129,550.8843	129,561.7019	7.13	723.76
GWO^66^	139,948.1	142,989.3	145,484.6	797.8	1766.2
SSO^65^	132,080.4	–	–	–	–
IABC^67^	129,862.0	129,895.0	–	40.8	4157.8
MFO^51^	129,708.1	–	–	–	–
Rao-1^65^	131,817.9	–	–	–	808.0
PSOGSA^44^	129,733.6	–	–	–	–
Rao-2^65^	131,490.7	–	–	–	804.6
MRao-2^65^	131,457.8	–	–	–	1160.3
ICBO^19^	135,121.6	–	–	–	–
MCSA^68^	129,873.6	–	–	–	–
Rao-3^65^	131,793.1	–	–	–	806.7
EWOA^69^	140,175.8	–	–	–	–
MSA^51^	129,640.7	–	–	–	–
FPA^51^	129,688.7	–	–	–	–
CS-GWO^64^	129,544.0	129,558.9	129,568.8	10.7	4252.5

Significant values are in bold.

### Case 2: OPF incorporating a quadratic cost function for conventional generators, along with the integration of solar and wind energy sources, can be articulated as follows:

Addressing the OPF challenge involves formulating a quadratic cost function for traditional generators, accounting for their operational costs. Additionally, the inclusion of solar and wind energy sources in this scenario introduces complexities related to their intermittent nature and variable outputs. The overarching goal is to optimize the power flow in the system while considering the unique characteristics and cost implications associated with both traditional and renewable energy sources.

This system is similar to the previous case study, incorporating renewable energy sources at various buses. Wind energy sources are placed at buses 18, 32, 36, 55, 104, and 110, while solar energy generation units are located at buses 6, 15, and 34.

The optimal solution for this case, obtained through the proposed SAWGA algorithm, is presented in Table 16 with a comparative study between the results of the algorithms WGA and the solutions obtained in reference^26^. These results demonstrate that SAWGA is a highly capable algorithm for optimizing and efficiently distributing loads in large, realistic power systems.

**Table 16:** Optimal results for Case 2.

Method	Min	Mean	Max	Std	Time (s)
SAWGA	**103,376.7409**	**103,495.5482**	**103,572.1029**	**44.60**	**623**
WGA	103,389.1573	103,507.1996	103,588.2463	104.70	625
BSA^26^	117,149.9833	120,443.2982	123,385.1256	1638.0949	–
DS^26^	110,992.4249	112,680.2902	114,787.7786	953.6529	–
DEEPSO^26^	103,407.6296	103,889.1446	104,507.4884	292.8782	–
MSA^26^	107,695.0619	111,205.0554	116,303.6361	1857.2167	–

Significant values are in bold.

### Ethical approval

This article does not contain any studies with human participants or animals performed by the author.

### Informed consent

Informed consent was not required as no human or animals were involved.

### Declaration of Generative AI and AI-assisted technologies in the writing process

The authors declare that they did not use AI-assisted technologies to create this article.

## Discussion

In the final discussion, the study has successfully implemented and assessed the enhanced Self-Adaptive Wild Goose Algorithm (SAWGA) in optimizing Economical-Environmental-Technical Optimal Power Flow (OPF) problems within traditional and modern energy systems. Leveraging adaptive search strategies and robust diversity capabilities, SAWGA, integrating four powerful optimizers, proves its efficiency. Applied to OPF models on IEEE 30-bus and 118-bus electrical networks featuring conventional thermal power units, solar photovoltaic (PV), and wind power (WT) units, SAWGA addresses the complexities introduced by renewable energy sources (RESs). The algorithm demonstrates superior performance in optimizing various objective functions, effectively managing OPF challenges, and consistently outperforming traditional WGA and other modern algorithms. Noteworthy attributes include its robust ability to achieve global or nearly global optimal settings for decision parameters, resulting in significant reductions in overall fuel consumption costs with faster and more efficient convergence. The findings highlight the substantial contributions of SAWGA in navigating the intricate landscape of OPF problems in the presence of RESs, reinforcing its role as a potent tool for the sustainable and efficient operation of power systems. On the one hand, considering the simulation times in the tables, especially based on Table 15, which represents simulation results for a large-scale energy system, it is evident that the proposed method exhibits a suitable optimization speed and time, particularly compared to the original WGA algorithm. The proposed SAWGA method significantly improves the final optimal results without introducing any specific complexity or additional simulation time compared to the original WGA algorithm.

## Conclusions

In conclusion, this study introduces the self-adaptive wild geese algorithm (SAWGA) as a novel and practical approach for addressing various optimal power flow (OPF) problems. We conducted a comprehensive comparison with four modern optimization algorithms (GOA, TEO, HHO, and HBA) and the traditional WGA, as well as the optimal results in the recent papers, showcasing SAWGA's superior efficiency. Our evaluation considered different objective functions within two different IEEE 30-bus and IEEE 118-bus electrical networks, incorporating wind turbine units (WTs) and solar photovoltaic units (PVs).

SAWGA demonstrates remarkable capabilities in optimizing diverse objective functions and effectively managing OPF challenges. The algorithm consistently outperforms traditional WGA and other modern algorithms, achieving global or nearly global optimal settings for decision parameters. The comparison of optimization results emphasizes SAWGA's superiority in total cost reduction and fast convergence.

Our future work will focus on developing a stochastic multi-objective model for the OPF problem in the presence of renewable power generations. This avenue promises to further enhance the applicability and robustness of optimization algorithms in addressing evolving challenges in power system management.

## Data Availability

All data generated or analyzed during this study are included directly in the text of this submitted manuscript. There are no additional external files with datasets.

## References

[1] Carpentier J (1962). Contribution to the economic dispatch problem. Bull. La Soc. Fr. Des. Electr..

[2] Shaikh MS, Raj S, Babu R, Kumar S, Sagrolikar K (2023). A hybrid moth–flame algorithm with particle swarm optimization with application in power transmission and distribution. Decis Anal J.

[3] Ghasemi M, Ghavidel S, Akbari E, Vahed AA (2014). Solving non-linear, non-smooth and non-convex optimal power flow problems using chaotic invasive weed optimization algorithms based on chaos. Energy.

[4] Ghasemi M, Ghavidel S, Aghaei J, Gitizadeh M, Falah H (2014). Application of chaos-based chaotic invasive weed optimization techniques for environmental OPF problems in the power system. Chaos Solitons Fractals.

[5] Farhat M, Kamel S, Atallah AM, Hassan MH, Agwa AM (2022). ESMA-OPF: Enhanced slime mould algorithm for solving optimal power flow problem. Sustainability.

[6] Biswas PP, Suganthan PN, Qu BY, Amaratunga GAJ (2018). Multiobjective economic-environmental power dispatch with stochastic wind-solar-small hydro power. Energy.

[7] Shaikh MS, Raj S, Ikram M, Khan W (2022). Parameters estimation of AC transmission line by an improved moth flame optimization method. J. Electr. Syst. Inf. Technol..

[8] Shaikh MS, Hua C, Jatoi MA, Ansari MM, Qader AA (2021). Application of grey wolf optimisation algorithm in parameter calculation of overhead transmission line system. IET Sci. Meas. Technol..

[9] Biswas PP, Suganthan PN, Amaratunga GAJ (2017). Optimal power flow solutions incorporating stochastic wind and solar power. Energy Convers. Manag..

[10] Guvenc U, Duman S, Kahraman HT, Aras S, Kati M (2021). Fitness-Distance Balance based adaptive guided differential evolution algorithm for security-constrained optimal power flow problem incorporating renewable energy sources. Appl. Soft. Comput..

[11] Ghasemi M, Ghavidel S, Ghanbarian MM, Gharibzadeh M, Azizi VA (2014). Multi-objective optimal power flow considering the cost, emission, voltage deviation and power losses using multi-objective modified imperialist competitive algorithm. Energy.

[12] Shaikh MS, Hua C, Hassan M, Raj S, Jatoi MA, Ansari MM (2022). Optimal parameter estimation of overhead transmission line considering different bundle conductors with the uncertainty of load modeling. Optim. Control Appl. Methods.

[13] Shaikh MS, Hua C, Raj S, Kumar S, Hassan M, Ansari MM (2022). Optimal parameter estimation of 1-phase and 3-phase transmission line for various bundle conductor’s using modified whale optimization algorithm. Int. J. Electr. Power Energy Syst..

[14] Duman S, Kahraman HT, Kati M (2023). Economical operation of modern power grids incorporating uncertainties of renewable energy sources and load demand using the adaptive fitness-distance balance-based stochastic fractal search algorithm. Eng. Appl. Artif. Intell..

[15] Hmida JB, Chambers T, Lee J (2019). Solving constrained optimal power flow with renewables using hybrid modified imperialist competitive algorithm and sequential quadratic programming. Electr. Power Syst. Res..

[16] Ullah Z, Wang S, Radosavljević J, Lai J (2019). A solution to the optimal power flow problem considering WT and PV generation. IEEE Access.

[17] Ali ZM, Aleem SHEA, Omar AI, Mahmoud BS (2022). Economical-environmental-technical operation of power networks with high penetration of renewable energy systems using multi-objective coronavirus herd immunity algorithm. Mathematics.

[18] Elattar EE (2019). Optimal power flow of a power system incorporating stochastic wind power based on modified moth swarm algorithm. IEEE Access.

[19] Bouchekara HREH, Chaib AE, Abido MA, El-Sehiemy RA (2016). Optimal power flow using an Improved Colliding Bodies Optimization algorithm. Appl. Soft Comput..

[20] Man-Im A, Ongsakul W, Singh JG, Madhu MN (2019). Multi-objective optimal power flow considering wind power cost functions using enhanced PSO with chaotic mutation and stochastic weights. Electr. Eng..

[21] Niknam T, Narimani MR, Aghaei J, Tabatabaei S, Nayeripour M (2011). Modified Honey Bee Mating Optimisation to solve dynamic optimal power flow considering generator constraints. IET Gener. Transm. Distrib..

[22] Salkuti SR (2019). Optimal power flow using multi-objective glowworm swarm optimization algorithm in a wind energy integrated power system. Int. J. Green Energy.

[23] Kahraman HT, Akbel M, Duman S (2022). Optimization of optimal power flow problem using multi-objective manta ray foraging optimizer. Appl. Soft Comput..

[24] Kathiravan R, Kumudini Devi RP (2017). Optimal power flow model incorporating wind, solar, and bundled solar-thermal power in the restructured Indian power system. Int. J. Green Energy.

[25] Riaz M, Hanif A, Hussain SJ, Memon MI, Ali MU, Zafar A (2021). An optimization-based strategy for solving optimal power flow problems in a power system integrated with stochastic solar and wind power energy. Appl. Sci..

[26] Duman S, Rivera S, Li J, Wu L (2020). Optimal power flow of power systems with controllable wind-photovoltaic energy systems via differential evolutionary particle swarm optimization. Int. Trans. Electr. Energy Syst..

[27] Chen G, Qian J, Zhang Z, Sun Z (2019). Multi-objective optimal power flow based on hybrid firefly-bat algorithm and constraints-prior object-fuzzy sorting strategy. IEEE Access.

[28] Duman S, Li J, Wu L, Guvenc U (2020). Optimal power flow with stochastic wind power and FACTS devices: A modified hybrid PSOGSA with chaotic maps approach. Neural Comput. Appl..

[29] Alanazi A, Alanazi M, Memon ZA, Mosavi A (2022). Determining optimal power flow solutions using new adaptive Gaussian TLBO method. Appl. Sci..

[30] Ghasemi M, Ghavidel S, Gitizadeh M, Akbari E (2015). An improved teaching–learning-based optimization algorithm using Lévy mutation strategy for non-smooth optimal power flow. Int. J. Electr. Power Energy Syst..

[31] Chen M-R, Zeng G-Q, Lu K-D (2019). Constrained multi-objective population extremal optimization based economic-emission dispatch incorporating renewable energy resources. Renew. Energy.

[32] Mouassa S, Althobaiti A, Jurado F, Ghoneim SSM (2022). Novel design of slim mould optimizer for the solution of optimal power flow problems incorporating intermittent sources: A case study of algerian electricity grid. IEEE Access.

[33] Venkateswara Rao B, Nagesh Kumar GV (2015). Optimal power flow by BAT search algorithm for generation reallocation with unified power flow controller. Int. J. Electr. Power Energy Syst..

[34] Ghasemi M, Davoudkhani IF, Akbari E, Rahimnejad A, Ghavidel S, Li L (2020). A novel and effective optimization algorithm for global optimization and its engineering applications: Turbulent Flow of Water-based Optimization (TFWO). Eng. Appl. Artif. Intell..

[35] Sarhan S, El-Sehiemy R, Abaza A, Gafar M (2022). Turbulent flow of water-based optimization for solving multi-objective technical and economic aspects of optimal power flow problems. Mathematics.

[36] Zahedibialvaei A, Trojovský P, Hesari-Shermeh M, Matoušová I, Trojovská E, Hubálovský Š (2023). An enhanced turbulent flow of water-based optimization for optimal power flow of power system integrated wind turbine and solar photovoltaic generators. Sci. Rep..

[37] Hassan MH, Elsayed SK, Kamel S, Rahmann C, Taha IBM (2022). Developing chaotic Bonobo optimizer for optimal power flow analysis considering stochastic renewable energy resources. Int. J. Energy Res..

[38] Chang Y-C, Lee T-Y, Chen C-L, Jan R-M (2014). Optimal power flow of a wind-thermal generation system. Int. J. Electr. Power Energy Syst..

[39] Kaveh A, Dadras A (2017). A novel meta-heuristic optimization algorithm: Thermal exchange optimization. Adv. Eng. Softw..

[40] Saremi S, Mirjalili S, Lewis A (2017). Grasshopper optimisation algorithm: Theory and application. Adv. Eng. Softw..

[41] Heidari AA, Mirjalili S, Faris H, Aljarah I, Mafarja M, Chen H (2019). Harris hawks optimization: Algorithm and applications. Futur. Gener. Comput. Syst..

[42] Hashim FA, Houssein EH, Hussain K, Mabrouk MS, Al-Atabany W (2022). Honey Badger Algorithm: New metaheuristic algorithm for solving optimization problems. Math Comput Simul.

[43] Ghasemi M, Trojovský P, Trojovská E, Zare M (2023). Gaussian bare-bones Levy circulatory system-based optimization for power flow in the presence of renewable units. Eng. Sci. Technol. Int. J..

[44] Zimmerman, R. D., Murillo-Sanchez, C. E., & Gan, D. Matpower. PSERC [Online] Softw. Available http://www.pserc.cornell.edu/matpower/ (1997).

[45] Ghasemi M, Ghavidel S, Rahmani S, Roosta A, Falah H (2014). A novel hybrid algorithm of imperialist competitive algorithm and teaching learning algorithm for optimal power flow problem with non-smooth cost functions. Eng. Appl. Artif. Intell..

[46] Ghasemi M, Zare M, Zahedi A, Trojovský P, Abualigah L, Trojovská E (2024). Optimization based on performance of lungs in body: Lungs performance-based optimization (LPO). Comput. Methods Appl. Mech. Eng..

[47] Ghasemi M, Aghaei J, Akbari E, Ghavidel S, Li L (2016). A differential evolution particle swarm optimizer for various types of multi-area economic dispatch problems. Energy.

[48] Shaikh MS, Ansari MM, Jatoi MA, Arain ZA, Qader AA (2020). Analysis of underground cable fault techniques using MATLAB simulation. Sukkur IBA J. Comput. Math. Sci..

[49] Ghasemi M, Rahimnejad A, Hemmati R, Akbari E, Gadsden SA (2021). Wild Geese Algorithm: A novel algorithm for large scale optimization based on the natural life and death of wild geese. Array.

[50] Brest J, Greiner S, Boskovic B, Mernik M, Zumer V (2006). Self-adapting control parameters in differential evolution: A comparative study on numerical benchmark problems. IEEE Trans. Evol. Comput..

[51] Mohamed A-AA, Mohamed YS, El-Gaafary AAM, Hemeida AM (2017). Optimal power flow using moth swarm algorithm. Electr. Power Syst. Res..

[52] Zimmerman RD, Murillo-Sanchez CE, Thomas RJ (2011). MATPOWER steady-state oper planning. Anal. Tools Power Syst. Res. Educ..

[53] Khunkitti S, Premrudeepreechacharn S, Siritaratiwat A (2023). A two-archive Harris Hawk optimization for solving many-objective optimal power flow problems. IEEE Access.

[54] Khunkitti S, Siritaratiwat A, Premrudeepreechacharn S (2022). A many-objective marine predators algorithm for solving many-objective optimal power flow problem. Appl. Sci..

[55] Abou El Ela AA, Abido MA, Spea SR (2010). Optimal power flow using differential evolution algorithm. Electr. Power Syst. Res..

[56] Sayah S, Zehar K (2008). Modified differential evolution algorithm for optimal power flow with non-smooth cost functions. Energy Convers. Manag..

[57] Kumari MS, Maheswarapu S (2010). Enhanced Genetic Algorithm based computation technique for multi-objective Optimal Power Flow solution. Int. J. Electr. Power Energy Syst..

[58] Ghasemi M, Zare M, Mohammadi SK, Mirjalili S (2024). Applications of Whale Migration Algorithm in Optimal Power Flow Problems of Power Systems.

[59] Kumar S, Chaturvedi DKK (2013). Optimal power flow solution using fuzzy evolutionary and swarm optimization. Int. J. Electr. Power Energy Syst..

[60] Sivasubramani S, Swarup KS (2011). Multi-objective harmony search algorithm for optimal power flow problem. Int. J. Electr. Power Energy Syst..

[61] Bhattacharya A, Chattopadhyay PK (2011). Application of biogeography-based optimisation to solve different optimal power flow problems. IET Gener Transm Distrib.

[62] Niknam T, Narimani M, Jabbari M, Malekpour AR (2011). A modified shuffle frog leaping algorithm for multi-objective optimal power flow. Energy.

[63] Narimani MR, Azizipanah-Abarghooee R, Zoghdar-Moghadam-Shahrekohne B, Gholami K (2013). A novel approach to multi-objective optimal power flow by a new hybrid optimization algorithm considering generator constraints and multi-fuel type. Energy.

[64] Meng A, Zeng C, Wang P, Chen D, Zhou T, Zheng X (2021). A high-performance crisscross search based grey wolf optimizer for solving optimal power flow problem. Energy.

[65] Hassan MH, Kamel S, Selim A, Khurshaid T, Domínguez-García JL (2021). A modified Rao-2 algorithm for optimal power flow incorporating renewable energy sources. Mathematics.

[66] El-Fergany AA, Hasanien HM (2015). Single and multi-objective optimal power flow using grey wolf optimizer and differential evolution algorithms. Electr. Power Comp. Syst..

[67] Bai W, Eke I, Lee KY (2017). An improved artificial bee colony optimization algorithm based on orthogonal learning for optimal power flow problem. Control Eng. Pract..

[68] Shaheen AM, El-Sehiemy RA, Elattar EE, Abd-Elrazek AS (2021). A modified crow search optimizer for solving non-linear OPF problem with emissions. IEEE Access.

[69] Nadimi-Shahraki MH, Taghian S, Mirjalili S, Abualigah L, Abd Elaziz M, Oliva D (2021). EWOA-OPF: Effective whale optimization algorithm to solve optimal power flow problem. Electronics.

